# Examining terror management theory in Ukraine: impact of air-raid alarms and explosions on mental health, somatic symptoms, and well-being

**DOI:** 10.3389/fpsyt.2023.1244335

**Published:** 2023-10-31

**Authors:** Stefan Stieger, David Lewetz, Svitlana Paschenko, Anton Kurapov

**Affiliations:** ^1^Department of Psychology and Psychodynamics, Karl Landsteiner University of Health Sciences, Krems an der Donau, Austria; ^2^Faculty of Psychology, Taras Shevchenko National University of Kyiv, Kyiv, Ukraine; ^3^Department of Psychology, Faculty of Natural Sciences, University of Salzburg, Salzburg, Austria

**Keywords:** terror management theory, mortality salience, mental health, somatic symptoms, experience sampling method, air-rail alarms, explosions, Ukraine

## Abstract

**Objective:**

This study sought to evaluate Terror Management Theory (TMT) assumptions about death awareness and its psychological impact in the context of a real-world war situation with high external validity. We examined if factors such as habituation to war circumstances and psychological resilience could buffer the effects on civilians’ anxiety, physical and mental health, and affect.

**Method:**

We implemented a pre-registered smartphone-based experience sampling method study over four weeks, with 307 participants (*k* = 7,824) living in war-affected areas in Ukraine whereby participants were regularly exposed to war situations, including air-raid alarms, explosions, and infrastructural problems.

**Results:**

The data indicated that war situations significantly increased anxiety, negatively impacting mental health, and raising somatic symptom severity. While habituation showed a mild buffering effect on these impacts, resilience did not.

**Conclusion:**

This real-world investigation supports TMT’s fundamental assumptions about death awareness and its psychological implications. However, even amidst the presence of real, life-threatening situations, the buffering effects of habituation were surprisingly minimal. This suggests that further exploration of TMT’s buffering factors in real-world scenarios is warranted.

## 1. Introduction

This study examines the profound psychological implications of human mortality, as postulated by the Terror Management Theory (TMT). According to TMT, awareness of death incites anxiety and influences overall well-being, potentially moderated by factors such as self-worth (1–3). However, previous research, despite supporting TMT, has primarily relied on experimental mortality salience interventions, thereby raising questions regarding external validity (1, 4). Furthermore, the proposed buffering effect of psychological aspects like self-worth and worldviews on death anxiety is inadequately explored (5, 6). Our research investigates these relationships within the authentic, distressing context of the war in Ukraine, utilising a longitudinal experience sampling design. This study focuses on the psychological impacts of war-specific situations, such as air-raid alarms and airstrikes (i.e., integral components of psychological warfare) on civilians. Historical accounts of psychological warfare have reported varying effects, from minimal psychological impacts to significant psychological and somatic symptoms. In light of this, we aim to assess the impact of these war-specific situations on anxiety, well-being, somatic symptom severity, and mental health, examining the potential buffering effects of habituation and resilience.

Our research is based on the understanding that mental health is an independent, unipolar dimension, not simply the absence of psychopathology but existing alongside mental disorders (7–9). Our research design, aiming for robust findings and enhanced ecological validity, includes a large sample size, daily assessments over four weeks, an examination of general physiological symptoms and mental health, daily anxiety level evaluations, the use of the affect grid for overall mood assessment, evaluation of war-specific experiences, investigation into habituation to war conditions, and consideration of resilience and general perceived stress as potential moderators.

### 1.1. Facing fear: terror management theory and its implications

It has long been assumed that confrontation with one’s own transience triggers a deep anxiety (10). Learning to deal with the finite nature of our lives is an important and defining issue for many. Based on this assumption, psychologists developed Terror Management Theory [TMT; Greenberg et al. (2)], which posits that awareness of death causes anxiety (reflected in the term “terror” in TMT), and this, in turn, affects psychological well-being. One possible cause for this dynamic comes from evolutionary theory (i.e., a basic motive of our existence is to survive). Nevertheless, a further assumption of TMT is that the link between death awareness and well-being is buffered (i.e., moderated) by other factors such as self-worth. For example, among people who have high self-worth, death awareness may less likely decrease well-being, compared with people who have low self-worth.

There is research supporting TMT (3). Predominantly, studies use different experimental designs to induce mortality salience in participants, such as reading death related essays, using subliminal primes, or watching videos, films, or slide shows [for a meta-analytic review, see (1)] to assess the impact on death anxiety or other psychological concepts including self-esteem, body satisfaction, materialism attitudes, or self-transcendent values (1). In recent years, there has also been an interest in the impact of mortality salience on mental health disorders [e.g., (11)] by assuming that the fear of death also contributes to the development and maintenance of mental health problems [for a review, see (12)]. For example, using a mortality salience experiment, researchers found that reminding participants of death led to compulsive handwashing in people with an obsessive-compulsive disorder (13), social anxiety in socially phobic individuals (14), and anxious behaviours in people with a panic disorder (15).

Nevertheless, research has also raised several concerns about TMT. First, almost all past research lacks external validity [i.e., generalisability to our everyday life; Burke et al. (1); for a similar reasoning, see (12)]. Second, one assumption of TMT is that psychological buffers such as self-worth and worldviews influence the link between death awareness and anxiety. For example, higher self-worth and higher motivation to defend one’s worldview are associated with a weaker association between mortality salience and anxiety. Interestingly, there is only very little empirical research which analysed this link in detail [for similar reasoning, see (6, 16)].

### 1.2. Air-raid alarms and airstrikes

Psychological warfare is an integral part of a war and is as old as humankind (17). In the last century, methods of psychological warfare have changed substantially, from airstrikes to newer forms of drone warfare (18). For example, airstrikes were used in World War (WW) I by Zeppelins, which dropped bombs on civilians [e.g., London; for a historical review, see (19)]. Airstrikes are associated with air-raid alarms, which warn the civil population of an impending airstrike. The main reason for these airstrikes was to put psychological pressure on the civil population due to the uncertainty of the time and place such airstrikes would take place. For example, airstrikes were used as an additional source of psychological warfare for a usually already distressed civilian population [e.g., due to deaths of family members; restrictions of everyday life; hunger; Linden (19) and Fegan (20)]. Whenever people are put under immense psychological stress for a certain time, people may develop somatic symptoms up to mental illnesses [e.g., anxiety disorders, panic attacks, post-traumatic stress disorders (PTSD), war syndromes; Jones and Wessely (21)].

### 1.3. Impact on mental disorders and health

Although there are reports of people developing severe psychological and somatic symptoms [e.g., (19); PTSD in soldiers: Johnson and Thompson (22)], other accounts describe the psychological impact of air-raid alarms and bombs on civilians as minor with extreme psychological reactions being rare [WWI: Anonymous (23); WWII: McLaughlin (24) and Vernon (25); Persian Gulf War: Sasson et al. (26); war in former Yugoslavia: Starcevic et al. (27)]. The reasons for this difference could be manifold. Propaganda and censorship in very early reports of WWI and WWII might have taken place by downplaying the psychological impact of the war situation on civilians. Furthermore, a conscious displacement of the psychiatric casualties after the war by re-labelling them becoming unrecognisable in hospital statistics have been described (19). Nevertheless, reports from more recent wars have found little evidence of a strong effect of air-raid alarms on mental health. For example, Sasson et al. (26) could not find any evidence that air-raid alarms raised the frequency of panic attacks in people with a panic disorder during the Persian Gulf War. Similar results have been found during the war in former Yugoslavia (27), such that no significant relationship between panic attacks and real danger was found. However, there was a direct impact of the war situation on feelings of anxiety, in line with one of the main assumptions of TMT. Therefore, panic attacks and fear induced by real danger might have different roots.

A further aspect of airstrikes is important to note. Some reports assume that air-raid *alarms* might have more impact on people’s fear and anxiety than the actual bombing itself (20). In contrast, based on contemporary literature, people may become accustomed to air-raid alarms such that alarms become a part of people’s life during wartime [e.g., (28)]. So, habituation of people to the war situation could be a reason why some authors did not find any substantial effects of the war situation on people’s psyche. This would be in line with one of the assumptions of TMT by postulating that psychological buffers can reduce the impact of a potential life threat on anxiety or well-being.

### 1.4. War situation and terror management theory: anxiety, physical, and mental health

From a theoretical point of view, air-raid alarms and explosions have the potential to activate mortality salience; that is: attention to one’s own death is made accessible according to TMT [e.g., (2, 29, 30)]. This usually heightens anxiety levels and compromises well-being. However, psychological buffers such as high self-worth can mitigate this effect. Self-worth can be developed through an individual sense of life, overcoming the physical self, and changing worldviews. Interestingly, there is very little real-life empirical evidence [e.g., (6)] on that assumption which has also been criticised in past research [e.g., (31)]. Nevertheless, as already evidenced, the war in Ukraine had a significant negative impact on the Ukrainian population, especially in terms of mental health and well-being for combatants (32), as well as civilians (32–37).

In the past several decades, psychological research has often focused on negative aspects of people’s psyche at a pre-clinical level, such as aggression, anxiety, racism, as well as mental disorders at a clinical level. However, this situation has changed since 1998 when Martin Seligman established work on Positive Psychology (38), which explicitly focuses on positive aspects of human life, such as mental health.

Mental health is not necessarily the absence of psychopathology. Several attempts have been made to operationalise mental health as a form of well-being and not the absence of mental disorder [for a review, see (39)]. A prominent approach, which is frequently used, is the concept from Keyes (9), who assumes that mental health (i.e., social, psychological, and emotional well-being) and mental disorder (e.g., panic disorder, major depressive episode, and generalised anxiety) constitute separate, although correlated, unipolar dimensions. Keyes found evidence that participants with a mental disorder can also have good mental health, termed “flourishing” [people with low mental health are “languishing”; see also Seligman (40)]. In a large U.S. adult population, Keyes found that only one fifth of participants were flourishing [i.e., had good mental health; Keyes (7, 9)]. In another study, it was found that adults with a mental disorder and moderate mental health (flourishing) functioned no worse than adults without a mental disorder but with low mental health [languishing; Keyes (8)]. Therefore, both concepts (mental health and mental disorder) are largely independent, but both are important when it comes to living a fulfilling life. This could also be a reason why some people develop a psychological disorder such as PTSD (low mental health), whereas others are rather resilient (high mental health) when faced with the same war-specific situations.

### 1.5. Research questions

This study aims to understand the psychological effects of war-specific situations on individuals and how factors such as resilience, habituation, and general stress may moderate these effects. Consequently, we present the following pre-registered research questions:

1. How do war-specific situations, such as air-raid alarms and explosions, and their resultant conditions (e.g., electricity outage, heating problems, water supply problems) impact anxiety, somatic symptom severity, mental health, and affect (both pleasure-displeasure and arousal-sleepiness scales)?
2. Is the association between war-specific situations and well-being (anxiety, somatic symptom severity, mental health, affect) moderated by generally perceived stress, resilience, habituation to air-raid alarms and explosions, and socio-demographic factors (age, sex)?

## 2. Materials and methods

### 2.1. Study design

In the present study, we want to test the assumptions of TMT by analysing the impact of war-specific situations (i.e., air-raid alarms, hearing airstrike explosions) on anxiety, well-being (affect), as well as somatic symptoms severity (as general indicators of mental illnesses), and mental health (flourishing) in a longitudinal design by using an Experience Sampling Method [ESM; Bolger (41) and Mehl and Conner (42)]. To analyse the assumptions of TMT in a real-word scenario with high ecological validity, we implemented the following design features: (1) a sufficiently large sample in order to have at least an 80% probability of replication if the hypothesis is true; (2) a longitudinal ESM study by assessing the concepts at hand during the everyday life of participants on a daily level for 4 weeks, which supports ecological validity by reducing biases (e.g., false memories, suppressed memories) and assessing the psychological concepts during the war-specific situations; (3) assessed severity of physiological/somatic symptoms in general (e.g., headaches, trouble sleeping, dizziness, chest pain) rather than specific clinical diagnosis; (4) assessed mental health (i.e., flourishing) in addition to somatic symptoms to also have a psychological view of well-being besides the physiological one; (5) assessed the general anxiety level each day as the most obvious emotion elicited by the war situation and a key concept in TMT (6); (6) assessed the general mood on that particular day, using the affect grid (43) as a quick measure assessing well-being along the dimensions of arousal-sleepiness and pleasure-displeasure; (7) assessed war-specific situations happened during each particular day (and previous night) because situations are frequent; (8) focused on the following core aspects based on the results of past research: Air-raid alarms and explosions (number, closeness) and their effects [i.e., electricity outage, heating problems, water supply problems; e.g., (26)]; (9) asked how participants are used to air-raid alarms and explosions at the beginning of the study and in the final questionnaire, to account for habituation [e.g., (28)]; and (10) assessed further moderating trait variables such as resilience (i.e., another psychological buffer against threat), general perceived stress, and socio-demographics (e.g., age and sex).

Based on this design, we were able to test several hypotheses. If the basic assumptions of TMT are true, then we should see heightened anxiety and lower well-being (i.e., more negative feelings), stronger psychosomatic symptom expression, and lower mental health after a day where war-specific situations took place (air-raid alarms, explosions, heating problems, water supply problems, and power outages). Furthermore, if a positive self-worth buffers against these threats, then resilient people and people who are already used to the war situation (i.e., habituation) should be less affected by these events. To control for the general stress level during the 4-week assessment phase, which might have influenced our measures (e.g., higher somatic symptom severity, lower pleasantness), we controlled for the general perceived stress in all our analyses.

### 2.2. Participants, recruitment, and power consideration

The study was pre-registered (https://osf.io/bnkjm).

We expected a small effect size (Pearson *r* = 0.1) for all research questions. First, we calculated a rough estimation for the minimum sample size for the multilevel approach by using the recommendation of Twisk (44). Based on a classical power analysis for a cross-sectional design, we needed *N* = 782 participants (bivariate correlation, *α* = 5%, minimum power = 80%, two-sided). Based on this calculation, we derived a minimum sample size for the multilevel approach. The duration of the study was set to 4 weeks (28 days). Therefore, the final sample size had to be at least *N* = 254 participants (28 repeated measurements, assumed intraclass correlation ICC = 0.30).

Second, we used a more elaborated approach to simulate power in multilevel designs by using the R-package *simr* (45) using the following assumptions: α = 5%, ICC = 0.30, number of retests = 28, number of participants = 300. Furthermore, to be on the safe side, we assumed small effect sizes for the standardised effects at Levels 1 and 2 and cross-level interactions (i.e., 0.1), and a random slope for Level 1 effects of 0.01 [small effect; for recommendations, see (46)]. Using 1,000 simulations and testing several combinations between the number of participants and a number of retests (assuming a dropout during data collection phase), we reached a power of 100.00% (95% CI = 99.63, 100.00) for 300 participants and 14 retests (assuming a conservative 50% non-response rate in the longitudinal part), and a power of 95.10% (95% CI = 93.57, 96.35) for 200 participants and 14 retests (for all calculated combinations, see https://osf.io/s2ypw/). To account for non-response and dropout (~10%–15%), we aimed for at least 200 participants.

Participants were recruited via Taras Shevchenko National University of Kyiv subject pools and personal contacts of the third and fourth authors (e.g., Facebook and Telegram). Through an information webpage generated by the used ESM platform ESMira (47), participants were provided with information about the procedures, inclusion criteria, objectives, and could download the ESMira app to their personal smartphone (Android or iOS) to sign in into the study in order to participate.

In sum, 460 participants joined the study and completed the demographic questionnaire right after enrolling to the study. 445 participants completed at least one single end-of-day questionnaire, and 311 participants stayed until the end of the study by also filling-in the final questionnaire. From the 445 participants filling-in the end-of-day questionnaire, 27 had to be excluded because having not completed the demographic and final questionnaire, or just completed the end-of-day questionnaire once. Furthermore, 9 participants had to be excluded because the stated age was <17 years. The difference between the remaining 409 participants and the 311 participants staying until the end of the study by filling-in the final questionnaire (*n* = 98) can be considered a classical dropout.

A dropout analysis revealed that there were no significant differences between dropouts and complete participants regarding sex (χ^2^ = 0.35, *p* = 0.84, φ = 0.03), age (*t* = −0.37, *df* = 379, *p* = 0.36, Cohen *d* = −0.04), whether they are already used to air-raid alarms and explosions (*t* = −1.41, *df* = 390, *p* = 0.08, *d* = −0.16), relationship status (χ^2^ = 0.82, *p* = 0.85, φ = 0.05), and the area the participant lives within Ukraine (χ^2^ = 3.50, *p* = 0.48, φ = 0.10).

This resulted in a final sample size of *N*_final_ = 307 participants who completed the final questionnaire and at least two end-of-day questionnaires (83.4% females, 0.3% diverse, *M*_age_ = 23.7, *SD*_age_ = 9.91, range = 17–54 years, for educational level distribution, see Supplementary material).^1^ All participants stated being of Ukrainian nationality except for one Russian citizen. On average, participants stated being already very used to air-raid alarms (demographic questionnaire: *M* = 71.0, *SD* = 23.44, range 0 to 100, Median = 73; final questionnaire: *M* = 71.0, *SD* = 25.53, range 0 to 100, Median = 76; *r* = 0.67; *t* = −0.23, *p* = 0.818). Moreover, 47.6% of the participants used Android smartphones, while 52.4% used Apple’s iPhones.

Regarding relationship status, 45.0% were single and/or living alone, 32.6% were in a relationship, 19.9% were married or in a partnership, 0.8% were divorced, and none stated being widowed (1.7% missing). The majority lived in the centre of Ukraine, with Kyiv as the capital (47.9%, final questionnaire 49.5%), 4.6% in the East (final questionnaire 2.9%), 23.5% in the North (final questionnaire 23.5%), 4.6% in the South (final questionnaire 3.9%), 9.1% in the West (final questionnaire 9.1%), and 10.4% did not state any area (final questionnaire 11.1%). We used this regional classification to assess the differences based on the proximity to the war zone (35) and averaged probability of air-raid alarms occurrence, meaning that they are rarer in Western regions in comparison to others (33). The comparison between the stated area in the demographic questionnaire and 4 weeks later in the final questionnaire shows that some participants probably had to move due to the war situation.

The project was approved by the ethics committee of the senior authors’ research institution (The Research Ethics Committee of the Faculty of Psychology of Taras Shevchenko National University of Kyiv, registration number 11-22/6).

### 2.3. Procedure

The data collection phase started in November 2022 and ended in January 2023 (recruitment on a rolling basis). For data collection and project administration of the ESM-part of the study, the open-source software *ESMira*^2^ (47) was used. ESMira is designed for scientific studies by offering a wide repertoire of functions and possibilities for scientific data collection (e.g., presentation and consent of the informed consent form, data encryption, data security, anonymous chat function, graphical feedback, anonymity through randomly generated codes, and anonymous reward option). ESMira was available for both Android and iOS operating systems.

Time-points to complete the surveys were predefined by the authors, and participants received a “bing” (i.e., signal, in-app reminder through a smartphone notification) through ESMira on their individual smartphone asking to complete a particular questionnaire. After clicking on that bing, the questionnaire opened automatically.

First, every participant answered demographic questions after having enrolled in the study (bing sent out automatically 1 min after enrolment by ESMira). This was done for two reasons. First, to assess data quality by comparing demographic information with the same questions from the final questionnaire 4 weeks later. Second, to introduce the functionality of bings to participants. Study administrators, as well as participants, quickly received feedback if everything worked as intended.

On a daily basis, participants completed an end-of-day questionnaire asking about events which happened on that particular day (e.g., air-raid alarms, heating problems), their mental health as well as somatic symptom severity. The bing was sent out at 8 p.m., but participants had the possibility of manually adapting this time-point in case of conflicting daily routines (e.g., night shifts). Overall, 111 participants used this option and changed the time schedule a total of 248 times during the study.

At the end of the 4-week assessment phase, a final cross-sectional questionnaire was sent to participants, which consisted of all demographic questions from the first questionnaire for data quality assessment. Furthermore, the following concepts were assessed by referring to the last 4 weeks: general somatic symptom severity [SSS-8; Narrow et al. (48)], perceived stress [PSS-4; Cohen et al. (49)], resilience [BRS; Smith et al. (50)], and mental health [MHC-SF; Keyes (9)]. Finally, participants were asked about general feedback and were instructed on how to get their rewards.

Participants who completed the demographic questionnaire, the final questionnaire, and at least one daily questionnaire were eligible for a 5 € reward (= 180 Hrywnja ₴), obtained through an individual anonymous reward code sent to the senior author (AK) via email. ESMira stated which questionnaire was missing for participants who did not complete all information. In the server-based admin-tool of ESMira, the reward code could be checked for validity, to avoid misuse. Because the ESMira-specific user-id and email-address never had to be used together, this procedure guaranteed that the anonymity of the participant is not breached and that the codes are valid [for more details, see (47)].

During the study, participants could view general as well as personalised statistics in graphical format directly in their ESMira app. The general statistic showed a bar chart of the frequency of completed end-of-day questionnaires over time on a daily basis (for example, see Supplementary Figure S1 – left panel). Furthermore, participants also got feedback about their individual data, i.e., their average mood (from the affect grid) over time, and a scatterplot showing their individual association between the activity level and anxiety level (see Supplementary Figure S1 – right panel). All participants used this option at least once (*M* = 18.3 times, *SD* = 17.43, Median = 11, range 1 to 90).

Because the study was anonymous and there was a large geographical deviation of participants, it was important to give participants support whenever a problem occurs. First of all, we used Frequently Asked Questions (FAQs) within ESMira to give participants support to questions which frequently arise due to the study’s design (e.g., “Where can I find my User-ID and is it anonymous?”; “I forgot to fill in the questionnaire yesterday. Should I make up for it today, a day later?”). Second, we used the built-in chat function of ESMira in order to give participants the possibility to send the study administrators a question anonymously. Furthermore, study administrators had the possibility to send participants (individual or groups of) a message, for example, if data were missing.

### 2.4. Measures

PSS-4, BRS, MHC-SF, and SSS-8 were translated to Ukrainian using a forward and backward translation procedure. Initially, AK and his colleague translated the items from English to Ukrainian (forward translation). Discrepancies between the two versions were resolved through the discussion between the two translators. Different translators, not aware of the original English version, translated items back into English (backward translation).

#### 2.4.1. Demographic questionnaire (cross-sectional)

Following the installation of ESMira and registration during the study, participants were asked (via bings) for basic demographic details [sex (male, female, diverse), age, nationality (Ukraine; other, please state), current relationship status, living in which region of Ukraine (East, Centre, North, South, West)]. Furthermore, it was asked about the habituation to air-raid alarms and explosions [“From your personal point of view, how much are you already used to air-raid alarms and explosions?” (0 = *‘not at all’*, 100 = *“already very much used to”*)].

#### 2.4.2. End-of-day questionnaire (longitudinal)

Participants were asked to complete a daily questionnaire concerning their mental health by using the Mental Health Continuum Short Form measure [MHC-SF; Keyes (9), Keyes et al. (51), and Żemojtel-Piotrowska et al. (52)] adapted for the use in ESM designs [i.e., asking about the current day instead of the last 4 weeks; see also (53)]. The MHC-SF consists of 14 items asking about particular feelings which were present on that particular day [e.g., “Over the course of today, how often did you feel … happy?” (*“never”, “once”, “twice”, “three times and more”*)] and assessed three dimensions of mental health: emotional, social, and psychological well-being.

Furthermore, we used the Somatic Symptoms Scale-8 [SSS-8; Narrow et al. (48)] to assess participants’ somatic symptoms severity as preliminary indicators of a possible mental illness. The SSS-8 is a reliable and valid short scale of the PHQ-15, which was developed within DSM-5 field trials (48). The SSS-8 consist of 8 items and asks about the severity of physiological complaints (e.g., stomach or bowel problems: 1 = *“not at all”*, 5 = *“very strong”*) in the last night and present day (“During the course of last night and today, how much did you feel affected by the following complaints?”).

We also used the affect grid [valence/pleasure, activation/arousal; Russell et al. (43)], which included: “How pleasant did you feel today on average?” [VAS: 0 = ‘*very unpleasant’*, 100 = ‘*very pleasant’*; “What was your average level of arousal today?” (VAS: 0 = ‘*drowsiness’*, 100 = ‘*high arousal’*)] followed by a question about the average anxiety level [“How was your average anxiety level today?” (VAS: 0 = ‘*no anxiety at all’*, 100 = ‘*high level of anxiety’*)], and several questions about the war situation on that particular day [“How many air raid alarms did you hear today?” (0, 1, >1); “How many explosions did you hear today?” (0, 1, 2, >2); “If there were explosions, from your subjective point of view, how far away was the closest explosion?” (VAS: 0 = ‘*very close’*, 100 = ‘*very distant’*); “Were you faced with an electricity outage today?” (‘*Yes’/*‘*No’*); “Were you faced with problems of heating your flat/house today?” (‘*Yes’/*‘*No’*); “Were you faced with water supply problems today?” (‘*Yes’/*‘*No’*)].

#### 2.4.3. Final questionnaire (cross-sectional)

To demonstrate the construct validity of our adapted mental health scale, we used the original mental health scale developed by Keyes (9) to assess participants’ stable, trait-like mental health (14 items, 6-point scale from 0 = *never* to 5 = *daily*). The same applied to the Somatic Symptom Scale (SSS-8), which we also used in its original form [8 items, 5-point scale from 0 = *not at all* to 4 = *very much*, (48)].

In addition, participants were asked to answer items regarding *perceived stress* (Perceived Stress Scale – PSS–4; Cohen et al. (49); 4 items, 5-point scale from 0 = *never* to 4 = *very often*) and *resilience* [Brief Resilience Scale – BRS; Smith et al. (50); 6 items, 5-point scale from 1 = *strongly disagree* to 5 = *strongly agree*]. With all measures, we asked participants to reflect on the last 4 weeks when answering the questions. All reliabilities were good to excellent (McDonald ω = 0.777 to 0.951, Supplementary Table S1 for details).

### 2.5. Statistical analyses

We used *R* (54) to conduct all statistical analyses using the *lme4* (55) and *sjstats* packages (56). Random-intercept, random-slope multilevel regression analyses were calculated in the first place because having a better fit than random-slope, fixed-intercept models. We encountered singular fit or non-convergence frequently; therefore, we successively excluded random-effects predictors one-by-one, starting with the least predictive, until there were no problems with model convergence or singular fit.

Then, we ran a baseline model without any predictors to calculate intraclass correlation coefficient (ICC) values (see Table 1). Next, we ran the multilevel model by simultaneously entering all Level 2 variables, i.e., demographics (age and sex), habituation, perceived stress level (PSS), and resilience (BRS). All Level 2 variables (except sex) were grand-mean centred (57, 58).

**Table 1:** Results of the multilevel analyses.

	Fixed						Random	
		*B*	SE	β	CI	*t*		*SD*
Somatic symptoms severity								
Intercept	β_00_	15.12	0.69	−0.14	−0.36, 0.08	22.00***	*r*_0*i*_	4.55
Within-person								
Air-raid alarms [1]	β_10_	0.37	0.11	0.06	0.03, 0.10	3.36***		
Air-raid alarms [>1]	β_20_	0.77	0.18	0.13	0.07, 0.19	4.41***		
Explosions [1]	β_30_	−0.29	0.25	−0.05	−0.13, 0.03	−1.17		
Explosions [2]	β_40_	−0.06	0.34	−0.01	−0.12, 0.10	−0.18		
Explosions [>2]	β_50_	0.33	0.23	0.05	−0.02, 0.13	1.41		
Electricity outage [Yes]	β_60_	0.01	0.16	> − 0.01	−0.05, 0.05	0.07	*r*_6*i*_	0.70
Heating problems [Yes]	β_70_	0.76	0.14	0.13	0.08, 0.17	5.26***		
Water supply pr. [Yes]	β_80_	0.33	0.16	0.05	0.00, 0.11	2.05*	*r*_8*i*_	0.66
Day of study	β_90_	−0.09	0.01	−0.12	−0.15, −0.09	−8.89***	*r*_9*i*_	0.02
Between-person								
Age	β_01_	−0.02	0.03	−0.04	−0.12, 0.05	−0.80		
Sex [female]	β_02_	0.49	0.74	0.08	−0.16, 0.32	0.66		
PSS-4	β_03_	1.89	0.39	0.24	0.14, 0.34	4.83***		
BRS	β_04_	−1.22	0.45	−0.13	−0.23, −0.04	−2.68**		
Habituation	β_05_	−0.01	0.01	−0.02	−0.11, 0.07	−0.49		
*R*^2^_conditional_ = 70%, *R*^2^_marginal_ = 13%, AIC = 35,282, BIC = 35,458, ICC = 65%								
Mental health								
Intercept	β_00_	15.87	1.21	−0.13	−0.34, 0.08	13.02***	*r*_0*i*_	7.96
Within-person								
Air-raid alarms [1]	β_10_	−0.32	0.19	−0.03	−0.06, 0.00	−1.69		
Air-raid alarms [>1]	β_20_	−0.27	0.31	−0.02	−0.08, 0.03	−0.87		
Explosions [1]	β_30_	−0.31	0.44	−0.03	−0.10, 0.05	−0.70		
Explosions [2]	β_40_	−0.60	0.60	−0.05	−0.16, 0.05	−0.99		
Explosions [>2]	β_50_	−1.06	0.41	−0.09	−0.17, −0.02	−2.63**		
Electricity outage [Yes]	β_60_	0.09	0.27	> − 0.01	−0.04, 0.05	0.35		
Heating problems [Yes]	β_70_	−0.89	0.28	−0.08	−0.13, −0.03	−3.16**	*r*_7*i*_	1.61
Water supply pr. [Yes]	β_80_	−0.45	0.29	−0.04	−0.09, 0.01	−1.56	*r*_8*i*_	1.46
Day of study	β_90_	−0.02	0.02	−0.01	−0.04, 0.01	−1.15	*r*_9*i*_	0.25
Between-person								
Age	β_01_	0.15	0.05	0.13	0.05, 0.22	3.21**		
Sex [female]	β_02_	2.42	1.32	0.21	−0.01, 0.44	1.84		
PSS-4	β_03_	−5.82	0.70	−0.39	−0.48, −0.30	−8.35***		
BRS	β_04_	0.33	0.81	0.02	−0.07, 0.11	0.41		
Habituation	β_05_	0.01	0.02	0.02	−0.07, 0.10	0.42		
*R*^2^_conditional_ = 73%, *R*^2^_marginal_ = 19%, AIC = 42,548, BIC = 42,724, ICC = 69%								
Anxiety								
Intercept	β_00_	28.20	2.34	−0.33	−0.50, −0.16	12.04***	*r*_0*i*_	15.26
Within-person								
Air-raid alarms [1]	β_10_	4.67	0.66	0.18	0.13, 0.23	7.02***		
Air-raid alarms [>1]	β_20_	5.45	1.06	0.21	0.13, 0.28	5.15***		
Explosions [1]	β_30_	7.80	1.53	0.29	0.18, 0.41	5.11***		
Explosions [2]	β_40_	12.37	2.07	0.47	0.31, 0.62	5.97***		
Explosions [>2]	β_50_	13.39	1.40	0.51	0.40, 0.61	9.58***		
Electricity outage [Yes]	β_60_	0.70	0.89	0.03	−0.04, 0.09	0.79		
Heating problems [Yes]	β_70_	3.13	0.94	0.12	0.05, 0.19	3.34***	*r*_7*i*_	5.45
Water supply pr. [Yes]	β_80_	1.39	0.91	0.05	−0.02, 0.12	1.52		
Day of study	β_90_	−0.21	0.05	−0.07	−0.10, −0.04	−4.24***	*r*_9*i*_	0.62
Between-person								
Age	β_01_	0.11	0.09	0.04	−0.02, 0.10	1.25		
Sex [female]	β_02_	5.14	2.42	0.19	0.01, 0.37	2.12*		
PSS-4	β_03_	5.85	1.27	0.17	0.10, 0.24	4.61***		
BRS	β_04_	−4.13	1.48	−0.10	−0.17, −0.03	−2.79**		
Habituation	β_05_	−0.10	0.04	−0.09	−0.15, −0.02	−2.63**		
*R*^2^_conditional_ = 42%, *R*^2^_marginal_ = 12%, AIC = 56,754, BIC = 56,903, ICC = 33%								
Affect grid – valence (negative vs. positive)								
Intercept	β_00_	54.43	1.84	0.11	−0.04, 0.26	29.64***	*r*_0*i*_	11.67
Within-person								
Air-raid alarms [1]	β_10_	−0.80	0.61	−0.03	−0.09, 0.02	−1.31		
Air-raid alarms [>1]	β_20_	−1.84	0.97	−0.08	−0.16, 0.00	−1.90		
Explosions [1]	β_30_	−2.95	1.40	−0.13	−0.25, −0.01	−2.10*		
Explosions [2]	β_40_	−3.01	1.93	−0.13	−0.29, 0.03	−1.56		
Explosions [>2]	β_50_	−4.87	1.29	−0.21	−0.32, −0.10	−3.77***		
Electricity outage [Yes]	β_60_	0.27	0.81	0.01	−0.06, 0.08	0.33		
Heating problems [Yes]	β_70_	−3.51	0.89	−0.15	−0.23, −0.08	−3.95***	*r*_7*i*_	6.39
Water supply pr. [Yes]	β_80_	−2.78	0.83	−0.12	−0.19, −0.05	−3.37***		
Day of study	β_90_	0.09	0.04	0.03	0.00, 0.06	2.19*	*r*_9*i*_	0.45
Between-person								
Age	β_01_	0.07	0.07	0.03	−0.02, 0.09	1.10		
Sex [female]	β_02_	−0.38	1.86	−0.02	−0.17, 0.14	−0.21		
PSS-4	β_03_	−8.58	0.97	−0.28	−0.34, −0.22	−8.89***		
BRS	β_04_	2.16	1.13	0.06	0.00, 0.12	1.90		
Habituation	β_05_	0.01	0.03	<0.01	−0.05, 0.06	0.20		
*R*^2^_conditional_ = 34%, *R*^2^_marginal_ = 12%, AIC = 56,543, BIC = 56,691, ICC = 30%								
Affect grid – arousal (low vs. high activation)								
Intercept	β_00_	57.20	1.83	−0.09	−0.24, 0.05	25.82***	*r*_0*i*_	10.22
Within-person								
Air-raid alarms [1]	β_10_	−0.79	0.65	−0.03	−0.09, 0.02	−1.21		
Air-raid alarms [>1]	β_20_	−0.59	1.03	−0.02	−0.11, 0.06	−0.58		
Explosions [1]	β_30_	1.46	1.50	0.06	−0.06, 0.19	0.98		
Explosions [2]	β_40_	−4.08	2.08	−0.17	−0.34, 0.00	−1.96*		
Explosions [>2]	β_50_	−1.47	1.39	−0.06	−0.18, 0.05	−1.06		
Electricity outage [Yes]	β_60_	1.33	0.87	0.06	−0.02, 0.13	1.52	*r*_6*i*_	3.14
Heating problems [Yes]	β_70_	−2.20	0.90	−0.09	−0.17, −0.02	−2.44*	*r*_7*i*_	4.60
Water supply pr. [Yes]	β_80_	−2.63	1.02	−0.11	−0.19, −0.03	−2.57*	*r*_8*i*_	7.60
Day of study	β_90_	0.03	0.03	0.01	−0.01, 0.03	1.00		
Between-person								
Age	β_01_	0.25	0.07	0.11	0.05, 0.16	3.77***		
Sex [female]	β_02_	3.83	1.86	0.16	0.01, 0.31	2.06*		
PSS-4	β_03_	−5.62	0.97	−0.18	−0.24, −0.12	−5.77***		
BRS	β_04_	2.60	1.14	0.07	0.01, 0.13	2.29*		
Habituation	β_05_	−0.01	0.03	<0.01	−0.06, 0.05	−0.15		
*R*^2^_conditional_ = 26%, *R*^2^_marginal_ = 7%, AIC = 57,611, BIC = 57,787, ICC = 26%								

Reference category for air-raid alarms and explosions was 0 (i.e., no air-raid alarms, no explosions) and for sex it was male. CI = 95% confidence interval; *β* = standardised B; PSS-4 = general perceived stress scale – 4; BRS = brief resilience scale; *p < 0.05, **p < 0.01, ***p < 0.001 (two-sided). ICC of the null model. When applying Bonferroni correction, all effects significant on *p* < 0.05 are not significant anymore.

Because within-subject reliabilities were lower for the mental health subscales compared to the overall score (Supplementary Table S2), and correlations of the subscales with the overall score were substantial (*r* = 0.89 to 0.95; see Supplementary Table S3), as well as the intercorrelations of the subscales were also high (*r* = 0.72 to 0.84; see Supplementary Table S3) we only used the overall mental health score for our main research questions [see also (53)]. Furthermore, we did not have any specific hypotheses regarding the subscales. We additionally included the day of filling-out the questionnaire to all our MLM analyses because values were slightly but significantly declining over time for somatic symptom severity (−0.11) and mental health (−0.02; see also Supplementary Table S4).

The random-intercept random-slope model is displayed below (full model before excluding Level 2 variables due to non-convergence or singular fit):

Level 1 (within person): (anxiety, symptom severity, mental health, activation, arousal)_ti_ = π_0i_ + π_1i_ Air-raid alarms[1]_ti_ + π_2i_ Air-raid alarms[>1]_ti_ + π_3i_ Explosions[1]_ti_ + π_4i_ Explosions[2]_ti_ + π_5i_ Explosions[>2]_ti_ + π_6i_ Electricity outage_ti_ + π_7i_ Heating problems_ti_ + π_8i_ Water supply problems_ti_ + π_9i_ Day of study_ti_ + *e*_ti_.

Level 2 (between persons): π_0i_ = β_00_ + β_01_ Age.cgm + β_02_ Sex(female) + β_03_ PSS.cgm + β_04_ BRS.cgm + β_05_ Habituation.cgm + β_06_ Day of study + *r*_0i_.

Level 2 (between persons): π_1i_ = β_10_ + *r*_1i_; π_2i_ = β_20_ + *r*_2i_; π_3i_ = β_30_ + *r*_3i_; π_4i_ = β_40_ + *r*_4i_; π_5i_ = β_50_ + *r*_5i_; π_6i_ = β_60_ + *r*_6i_.

We used *R*^2^_GLMM_ (59, 60) as a measure of explained variance, which can be interpreted like the traditional *R*^2^ statistic in regression analyses. *R*^2^_marginal_ represents the proportion of variance explained by the fixed factors alone, and *R*^2^_conditional_ the proportion of variance explained by both fixed and random factors. Additionally, following Nakagawa and Schielzeth (60), we also included AIC and BIC as information criteria indices.

For research question 2, we calculated random-intercept, fixed-slope multilevel models because all full random models again failed to reach convergence. Because of the large number of interaction terms when including all Level 2 variables into a single model (eight Level 1 variables, five Level 2 variables), we decided to analyse interactions by only including one Level 2 variable at a time resulting in 5 multilevel models. Furthermore, because of the still large number of single results, we presented only the effect size of each predictor together with the level of significance (for the values, see Supplementary Table S5).

We applied Generalisability Theory Analysis [GTA; Brennan (61) and Shrout and Lane (62)] by using the *multilevel. Reliability* function in the *psych* package in *R* (54) to analyse the within-person as well as inter-individual reliability. Missing values were, in general, very rare (< 0.5%) or even not possible due to a forced-response design (e.g., MHC-SF in the longitudinal phase) and were replaced by using the nearest neighbour method (except demographic variables). Because the assessment of habituation to air-raid alarms and explosions from the demographic questionnaire and the final questionnaire were highly correlated with no mean difference (*r* = 0.67; *t* = −0.23, *p* = 0.818), we calculated a mean score and used this one in all further analyses. Finally, there was only one person who self-stated being diverse regarding their gender; we, therefore, excluded this person from further analysis.

All data, analysis scripts, and materials can be found online at https://osf.io/s2ypw/.

## 3. Results

### 3.1. Deviation from the pre-registration

First, although we stated in the pre-registration that we will include all longitudinal questionnaires from all participants, we decided to exclude participants who completed only a single questionnaire out of the possible 28 questionnaires (*n* = 24; 0.3% of cases) because of non-compliance. All 24 did not complete the final questionnaire and, as such, were considered dropouts. Second, we did not include area where participants lived in Ukraine in the analyses because many (12.7%) moved during the 4-week assessment phase (i.e., area after 4 weeks was different from the one stated in the demographic questionnaire) or misclassified the area where they live. Third, participants stated that there were no explosions in 87% of cases; therefore, we excluded the question of closeness of the explosion from further analysis (*n* = 670; 8.6% of cases; *M* = 46.7, *SD* = 27.7, Median = 45, range 0 to 100). Finally, although we stated in the pre-registration we were going to use VAS from 1 to 100, due to technical reasons, we had to use a VAS from 0 to 100 (i.e., faulty hard coded definition in ESMira).

### 3.2. Validity check and reliabilities

Participants stated their demographics at two points during the study, at the beginning and in the final questionnaire. All demographics were highly consistent. Responses for participants’ sex showed only one deviation, and for age, only one deviation larger than 1 year. Relationship status was also highly consistent (Kappa κ = 0.90), showing some inconspicuous changes (*n* = 19 participants changed their current relationship status during the 4-week assessment phase).

Our measure of mental health in the daily questionnaire was substantially correlated with mental health (*r* = 0.70, *p* < 0.001) and moderately correlated with somatic symptoms stated in the final questionnaire in line with past research [4-week time frame, *r* = −0.23, *p* < 0.001, (63), *r* = −0.27, (53), *r* = −0.40]. Furthermore, daily mental health was correlated with resilience (*r* = 0.20) and perceived stress (*r* = −0.46) in the final questionnaire (across all data points; all *p*s < 0.001). Similarly, somatic symptom severity assessed in the daily questionnaire was substantially correlated with the somatic symptoms (*r* = 0.69, *p* < 0.001) and moderately correlated with mental health (*r* = −0.24, *p* < 0.001) in the final questionnaire. Furthermore, somatic symptom severity on a daily basis was correlated with resilience (*r* = −0.26) and perceived stress (*r* = 0.33) in the final questionnaire (across all data points; all *p*s < 0.001). All results speak for the criterion and construct validity of the daily mental health and somatic symptoms measure used in the current study. For all intercorrelations between Level 2 measures and separately for males and females, see Supplementary Table S3.

Internal consistencies of all trait-level instruments used were good to excellent (Cronbach α = 0.783 to 0.952; McDonald ω = 0.781 to 0.952, see Supplementary Table S1 for details). For the longitudinal Level 1 measurements, we calculated within- and between-subject reliabilities. Due to the large sample size (which substantially slows down the calculation procedure in *R*) as well as to analyse the reliability equivalence between the Operating Systems (OS) Android and iOS smartphones (i.e., graphical display of questionnaires slightly differs because of the different basic designs of the OS), we calculated the reliabilities for both OS separately. Within-person (*R*_C_ = 0.60 to 0.87) and between-person (*R*_kR_ = 0.94 to 0.98) reliabilities were good to excellent for mental health as well as somatic symptom severity (see Supplementary Table S2 for details), and differences between OS were negligible (maximum difference = 0.02). This suggests reliable assessment of both within-person changes and inter-individual differences as well as across different OS (64).

### 3.3. Descriptive statistics/preliminary analyses

**Figure 1:**
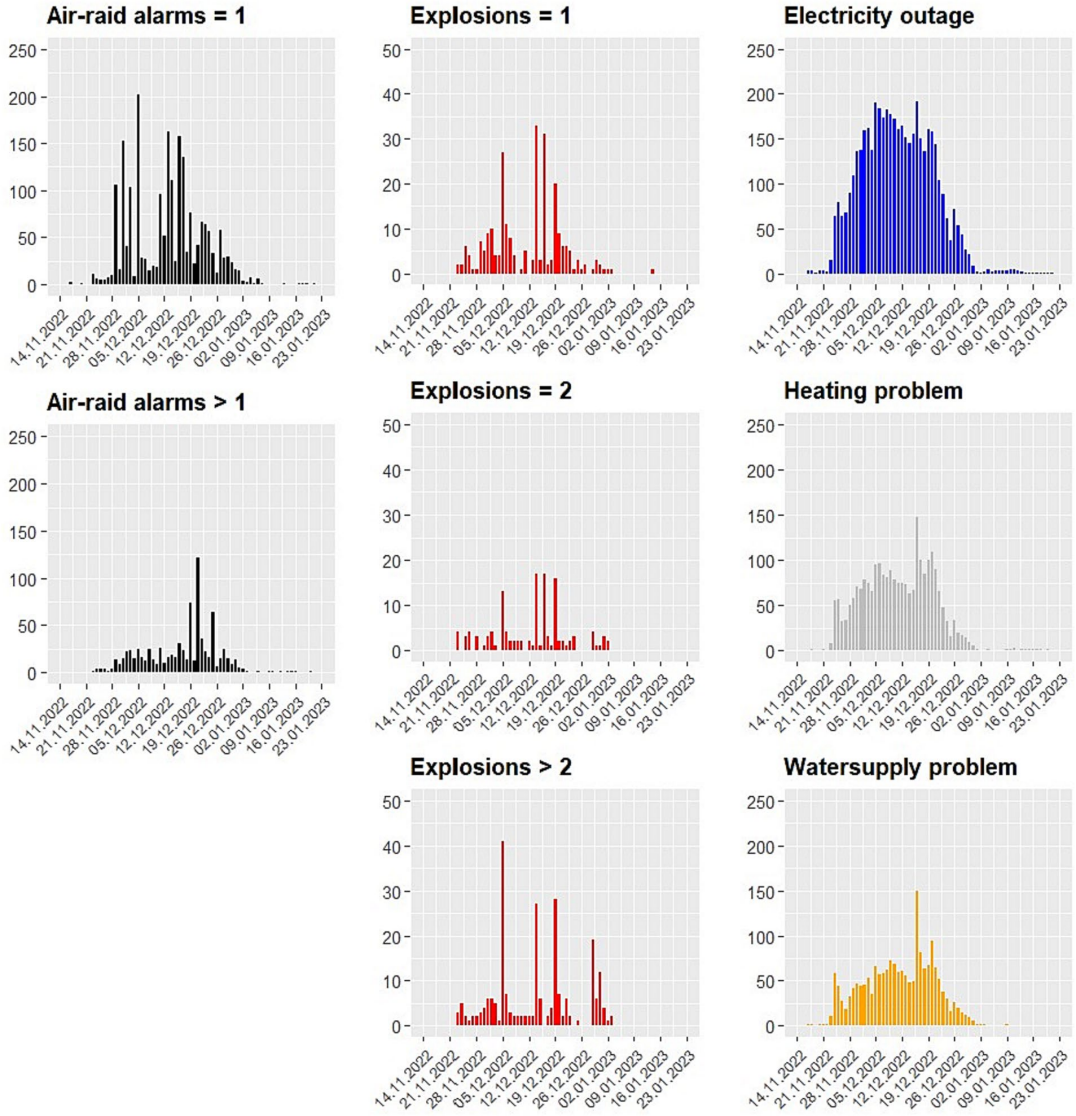
Frequency of reported war-specific situations experienced by participants during the assessment phase.

A substantial percentage of participants overall were affected at least once by our war-specific indicators during the 4-week assessment phase (air-raid alarms: 83.1% of participants; explosions: 57.5%; electric outages: 85.8%; heating problems: 71.1%; water supply problems: 69.7%; see Figure 1 for the frequency over time). This is also in line with independent statistics collected during the assessment phase (November 2022 to January 2023), 2,144 air-raid alarms have been triggered throughout the Ukraine, and 790 explosions.^3^

During the 4-week assessment phase, participants had, on average, an anxiety level of 34.2 (Median = 30, range 0 to 100) with a large standard deviation (*SD* = 26.4), indicating that anxiety strongly varied over time. In 27.2% of daily questionnaires (2,117 out of 7,665), participants stated that there was an air-raid alarm on that particular day (more than one air-raid alarm: 10.4%; 1.7% missing). In 3.2% of days, they reported hearing a single explosion on that day (two explosions: 1.6%, more than two explosions: 4.1%; 3.5% missing). In 58.8% of cases, they reported an electricity outage (1.5% missing), in 31.1% a heating problem (1.6% missing), and 24.3% a water supply problem (1.8% missing). Although some of the percentages of war-specific situations appear low (e.g., explosions), these figures are around 0% in countries *without* a war situation.

Because participants frequently used the graphical feedback function in ESMira (see Supplementary Figure S1 for an example), the question arose as to whether or not this could constitute an intervention effect itself on our dependent measures [i.e., reactivity; e.g., (65)]. For example, an individuals’ mood levels may decline over time as a result of that individual seeing graphical feedback that their own self-reported mood is declining over time. We calculated simple fixed-effects multilevel models with the number of graphics viewed and the day of the study, and the interaction between both onto the dependent variable (somatic symptoms, mental health, anxiety, pleasure, and arousal). To avoid problems of multicollinearity, the *number of graphics* was grand-mean centred and *day of study* person-mean centred. If there is an intervention effect, we would expect that a possible influence on our dependent measures changes over time, i.e., there should be an interaction effect between *total number of graphics* viewed and the *day of study*. Indeed, we found three significant intervention effects [for mental health (β = 0.02), anxiety (β = −0.03), and pleasantness (β = 0.02)], although all had a small effect size (see Supplementary Table S4 for details).

### 3.4. Research question 1: what impact do air-raid alarms and explosions and resulting situations thereof have on anxiety, mental health, severity of somatic symptoms, and well-being (i.e., pleasantness and activation)?

**Figure 2:**
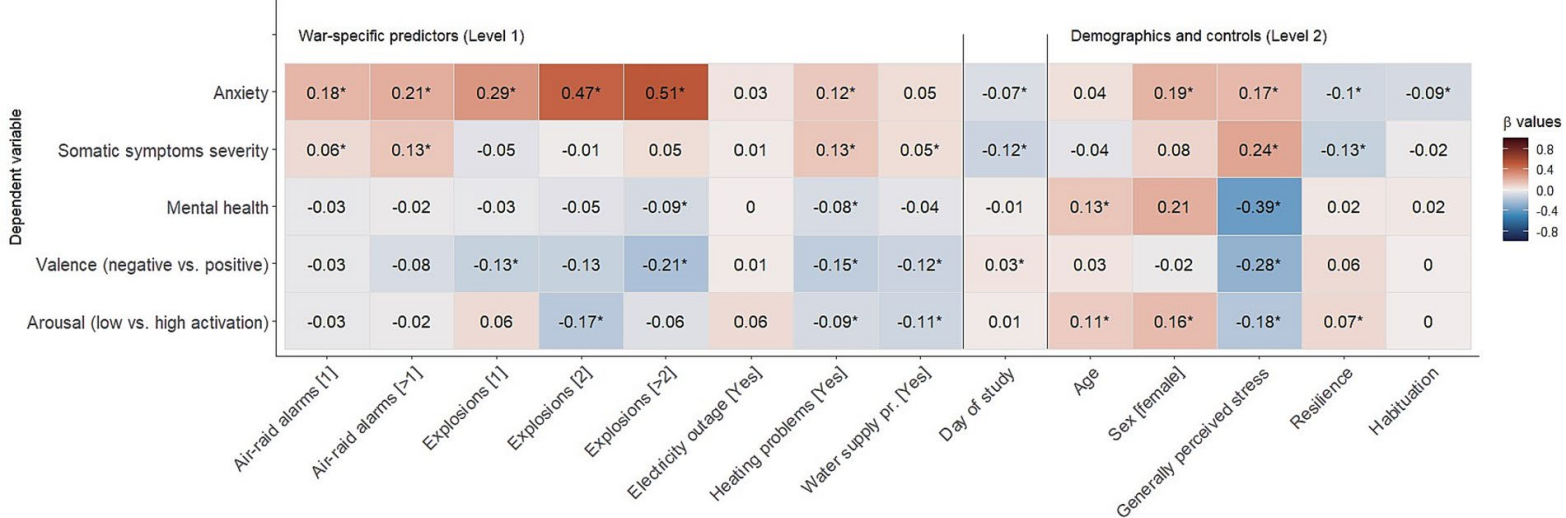
Results of the multilevel model (Standardised β values; * *=* significant).

As can be seen in Table 1 and Figure 2, results are manifold. Strongest effects of the war situation were found with anxiety levels in line with TMT. Explosions and air-raid alarms had a substantial effect, followed by heating problems (assessment phase was during winter months of January and February). Electricity outages and water supply problems had no significant effect (see Table 1). Women and participants with a high general perceived stress had higher anxiety values, while resilient participants had lower anxiety scores, as expected. Habituation only mildly buffered against feelings of anxiousness.

When it comes to somatic symptoms, air-raid alarms significantly raised the severity of symptoms, but explosions and electricity outages had no substantial effect. Heating and water supply problems again raised symptom severity. No sex-specific effects and effects of habituation were found. As expected, general perceived stress was positively associated with symptom severity and negatively associated with resilience. Despite being statistically significant, the effect sizes of the war-specific situations [Mean |β| = 0.06, i.e., very small effect; Funder and Ozer (66)] were substantially smaller than for anxiety (Mean |β| = 0.23, i.e., medium effect).

A similar picture compared to somatic symptoms emerged for mental health where again small effect sizes have been found for the war-specific situations (Mean |β| = 0.04, i.e., very small effects). Having experienced heating problems and more than two explosions on a particular day lowered mental health significantly, but air-raid alarms, electricity outages, and water supply problems had no significant impact. Older participants were slightly more mentally healthy, and participants with generally large perceived stress had lower mental health scores which was even the strongest effect (β = −0.39; see Table 1).

Similar results were found for the other indicator of well-being; that is: the judgement of the valence (negative vs. positive) of one’s well-being on a particular day. Explosions, heating problems, and water supply problems were significantly associated with lower positivity. Regarding trait variables, only the perceived general stress showed any significant association and a large effect size. In general, again, effect sizes were small across demographics and trait variables (Mean |β| = 0.11, i.e., small effect).

Finally, war-specific situations were associated with reduced activation among participants (Mean |β| = 0.08, i.e., small effect). In general, older participants, and females, were slightly more activated than younger participants, and men, respectively. More generally, stressed participants had lower activation, and resilient people were slightly more activated than low-resilient people. Again, effects sizes were rather small-to-medium.

In sum, the following general results were observed (Figure 2): war-specific situations had the strongest effects on experienced anxiety (|β|s = 0.03 to 0.51), followed by mental health (|β|s = 0.01 to 0.09) and somatic symptom severity (|β|s = 0.01 to 0.13), although anxiety showed more variability over time within participants (ICC = 33%) than mental health (ICC = 69%) and somatic symptoms (ICC = 65%) (Table 1) suggesting these concepts were more stable longitudinally. These results underline one of the main assumptions of TMT that mortality salience heightens anxiety and can also impact other psychological concepts associated with well-being (physical and mental health, affect).

### 3.5. Research question 2: are there any moderators?

**Figure 3:**
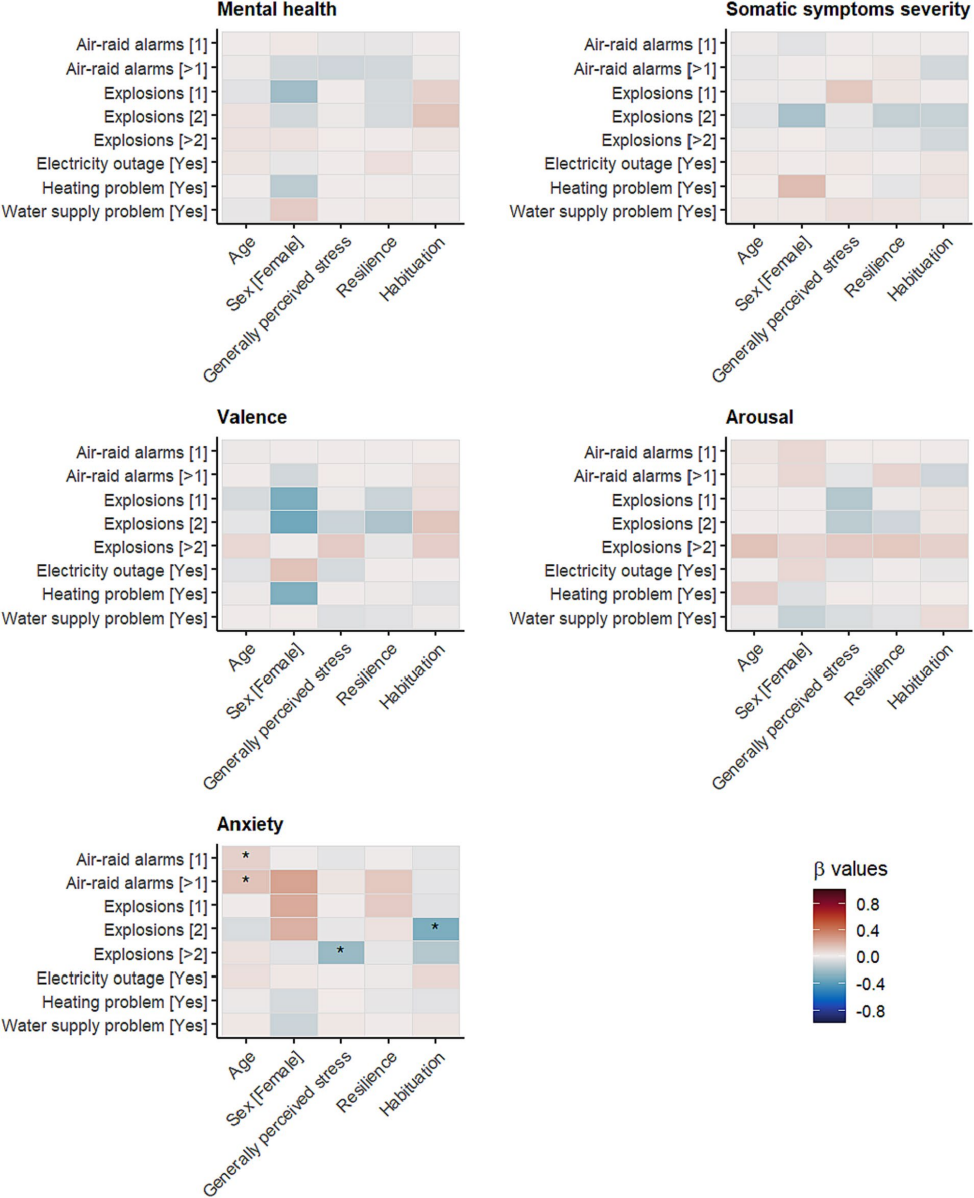
Standardised cross-level interaction effects (Bonferroni corrected significance level: *p < 0.00125).

As can be seen from Figure 3, having low general perceived stress, higher resilience, and habituation to the war situation (all Level 2) did not appear to have an impact on the association between war-specific situations and our dependent measures: somatic symptom severity, mental health, and well-being (valence, arousal). This also applies to demographic differences regarding participants’ sex and age. The only significant interactions were found for anxiety, where older participants stated higher anxiety due to air-raid alarms than younger participants (β = 0.09 and 0.14, respectively, Supplementary Table S5), although effect sizes were small. Significant effects were also found for the impact of explosions, yet this is counterintuitive because reporting more than two explosions and higher general perceived stress was associated with lower anxiety scores when compared to the group without any explosions on that particular day (β = −0.24; Figure 3). This might be due to the rather low frequency of participants having experienced more than two explosions on a particular day where no association was found (frequency: 4.1%; Supplementary Figure S2). In line with TMT, a significant interaction between habituation and anxiety was found when faced with explosions, such that habituation and anxiety were negatively associated among people who experienced two explosions compared to people who experienced no explosions (i.e., low association between habituation and anxiety; effect size of the difference in associations: β = −0.31). Interestingly, this effect was weaker and not significant among participants who experienced *more than two* explosions (β = −0.17; ns) although this might be due to the low frequency of cases (frequency: 1.6%, Supplementary Figure S3).

### 3.6. Explorative analyses: is mental health a further psychological buffer?

**Figure 4:**
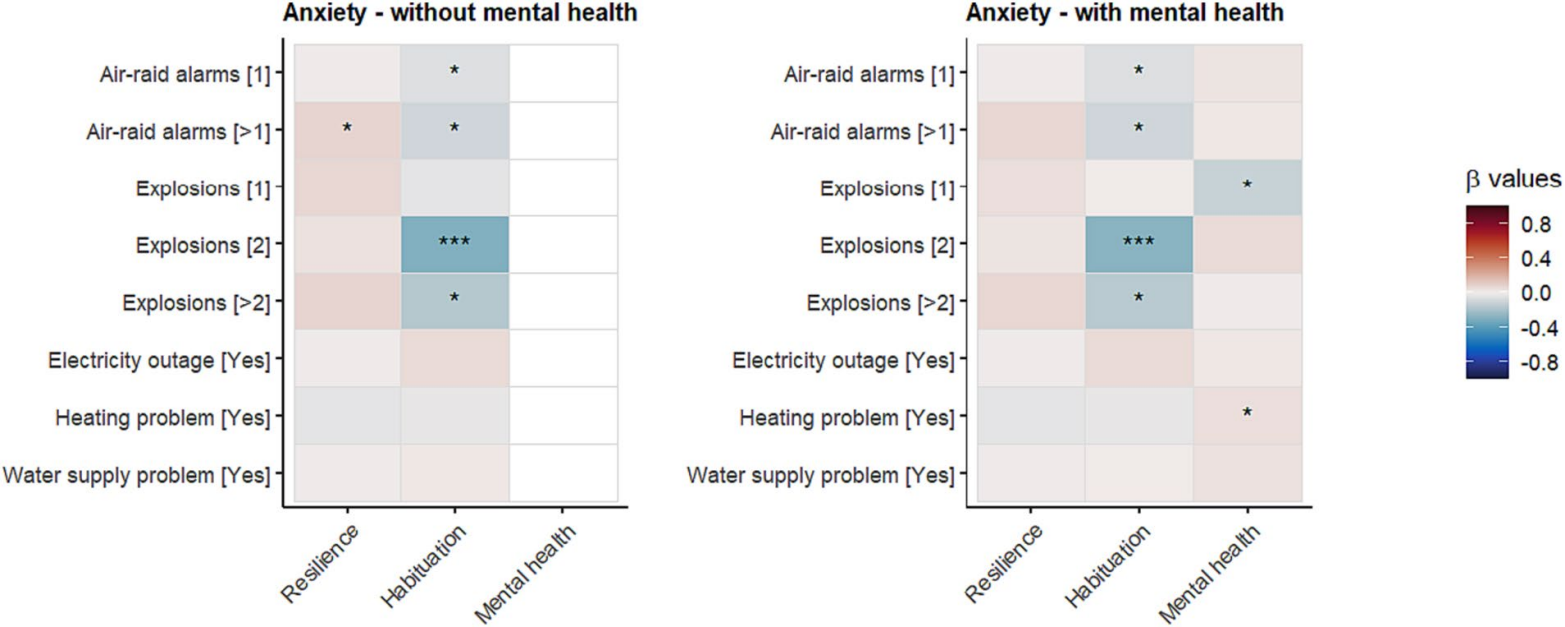
Impact of psychological buffers (resilience, habituation, and mental health) on the association between war-specific situations and anxiety. *p < 0.05; ***p < 0.001; When applying Bonferroni correction, only the effect on habituation and having experienced two explosions is significant (see also Figure 3).

Mental health could act as a psychological buffer against the impact of war-specific situations on anxiety. If positive mental health acts as a resilience factor based on the assumptions of TMT, then we would expect that positive mental health buffers against the threat of war-specific situations (i.e., mental health is a moderating factor). Because we did not pre-register this particular research question, we present the results as explorative. As can be seen from Figure 4 (for details, see Supplementary Table S6), although mental health showed some moderating effects between explosions/heating problems and anxiety, when applying Bonferroni correction, these effects are not significant anymore.

## 4. Discussion

In the present study, the following main results were found. As expected, based on the assumptions of TMT, the war situation (air-raid alarms, explosions) had an immediate effect on perceived anxiety (27, 67). Also, as expected, situations resulting from the direct threat of war which are less immediately life-threatening (e.g., heating problems) had a lesser impact on perceived anxiety. In contrast to anxiety, somatic symptom severity was less affected by the war situations (only air-raid alarms) as well as mental health; specifically, having heard more than two explosions significantly reduced mental health, although effect sizes were substantially smaller than for anxiety. Explosions had a similar effect on affect, such that they reduced mood and slightly reduced arousal, but again effect sizes were smaller than those for anxiety. In general, situations stemming from war situations had only a small impact. Electricity outages had no significant effect at all, and heating and water supply problems only had little influence on anxiety. Overall, the findings lend support to our first research question and one of the basic assumptions of TMT. Furthermore, all these effects appeared to be independent of participants’ age and sex as well as general perceived stress-level, resilience, and habituation to the war situation, except for some minor effects found for anxiety. This, in part, supports our second research question that habituation and/or resilience buffers against life-threatening war situations.

As expected, and also in line with the assumptions of TMT, war-specific situations elicited anxiety and negative valence. Interestingly, war-specific situations *lowered* the activation level of participants, which might be counterintuitive. Fear and threat elicit action to deal with threats – often referred to as the “fight, flight, or freeze” reaction (68) – may explain this result. In the present study, the threat of war may lead to ‘freeze’ reactions which can manifest themselves as withdrawal, freezing in place, or avoiding certain situations (e.g., staying at home, do not waste personal resources, endure the situation). This is understandable since the war situation cannot easily be resolved by the civil population (e.g., avoiding water-supply problems due to bombings).

Also of interest is that electricity outage was not a significant predictor of any outcome; that is: electricity outage did not appear to impact participants’ lives to a degree through which they experienced negative psychological or physical health. Furthermore, habituation was also of low predictive power and only reached statistical significance for anxiety; participants appeared to be used to air-raid alarms and explosions, which may buffer them against the impact of war-specific situations (28). This is in line with one of the basic assumptions of TMT, although effects were weak in our study. However, past research operationalised “psychological buffer” as having self-worth and stable world views, rather than habituation and resilience as defined in the present study, which may explain the differences between the present study and prior work.

Mental health seems to be largely different from mental illness when defined as somatic symptom severity, since we observed only a small-to-medium negative association [*r* = −0.27; see Supplementary Table S3; Keyes (7, 9)]. The pattern of effects was very similar for air-raid alarms and explosions, although effect sizes were small. Furthermore, although more resilient participants were less anxious, had less somatic symptom severity, had higher mental health, and felt more positive than less resilient participants, they appeared to be equally affected by war situations. This suggests that being more resilient may not buffer against war-specific situations, although again, effect sizes were small. This is also in line with the exploratory analysis. Only habituation to the war situation had some buffering effects by reducing the association between explosions and anxiety, such that the higher the habituation, the less likely explosions elicited anxiety (see also Supplementary Figure S3).

These results largely underline the assumptions of the TMT; specifically that a potential threat to one’s life leads to heightened anxiety and lower well-being (higher somatic symptom severity, lower well-being, lower positivity; Research question 1). Nevertheless, the mortality salience intervention was in the present study a real life-threat in contrast to the often-used mortality salience instruction of experimental studies (e.g., reading texts about death); we would therefore have expected stronger effects based on the real situation. A result less in line with TMT was that being resilient, or habituated to the war situation, did not substantially mitigate the impact of mortality salience on our dependent measures.

It has been argued that air-raid alarms might have a larger impact than the actual bombing (20) or hearing explosions thereof. Our results found that this appeared to be true for symptom severity, but that explosions (rather than air-raid alarms) had a larger impact on all other outcomes (anxiety, affect grid, mental health), contrary to this theory.

In sum, our study is consistent with past research in two primary ways. First, it is consistent with findings from WWI (23), WWII (24, 25), Persian Gulf War (26), and former Yugoslavia (27), suggesting that the civil population is overall rather resilient to indirect war-specific situations (i.e., no direct contact with combat operations on the front lines) when it comes to their mental health and physiological symptom severity. Second, our study largely supports the assumptions of TMT, whereby effects on anxiety were largest, followed by impact on well-being as well as symptom severity. However, the assumption of psychological buffering effects (habituation and resilience) could only be confirmed for habituation, but not resilience or mental health, and effects were weaker compared to the direct link between mortality salience and anxiety.

### 4.1. Limitations

Although our design had several advantages compared to previous studies, our study also has several limitations. First of all, we assessed only a time-frame of 4 weeks between December 2022 and January 2023, which might not be representative of the general war situation in Ukraine (i.e., habituation since the start of the war might have already taken place). This is also of relevance when it comes to the long-term effects of war situations, which are often relevant for severe psychological problems such as PTSD. Although we found rather weak effects of war-specific situations on well-being on a daily basis, these weak effects might accumulate over time, leading to severe symptoms later on. Second, although sufficiently powered, our sample was majority female (83.4%), so findings may not generalise to males; however, at the time of the data collection, compulsory military service was in force for males. Furthermore, participants were on average younger (*M*_age_ = 23.7 years) compared to a community-based sample and so findings may not generalise to older adults. Third, we asked participants about anxiety in general, whereas, in TMT research, participants are often asked specifically about *death* anxiety. Nevertheless, effects on anxiety in our study were substantial and similar to those observed in past research using a general anxiety scale (69). Fourth, although our design has high ecological validity, concepts were assessed at the end of the day by retrospectively remembering what happened on that particular day and how one felt. Asking questions about anxiety or well-being directly after events such as air-raid alarms and explosions, such as by using an event-based sampling schema, might have improved ecological validity but would have been ethically problematic. Furthermore, TMT also assumes that a link between mortality salience and anxiety is distal, such that it does not establish itself immediately but only after a couple of minutes; therefore, in experiments, often filler-tasks are used [see Juhl and Routledge (6) for a discussion about proximal and distal measurement of anxiety after mortality salience in TMT research]. Moreover, immediate assessment after the event might even be detrimental.

### 4.2. Future directions

In the present study, we analysed the negative effects of a war situation on the civil population in Ukraine. Future research might analyse possible positive effects when the war is over. The Post Traumatic Growth (PTG) theory assumes a positive effect due to potentially life-threatening situations which have ended. The effect is defined as “positive psychological change experienced as a result of the struggle with highly challenging life circumstances” (70). It is assumed that PTG is more than just a return to the psychological state prior to the war situation; instead, PTG involves positive changes in self-perception, enhanced levels of functioning, and developing closer relationships with others (71).

## 5. Conclusion

In the present study, we used a sufficiently large sample to allow us to detect small effect sizes. This allowed us not only to judge if an effect is significant, but also *how important* the effect is for the assumptions of TMT. We applied a sophisticated design (4-week ESM) by focusing on physiological (symptom severity) and psychological variables (mental health, anxiety, and affect) to analyse the impact of war-specific situations (air-raid alarms and explosions) and situations that arise from this (electricity outage, heating problems, and water supply problems). Therefore, the study had high ecological validity to test the assumptions of TMT.

The present study seems to be one of the few studies analysing TMT in real-life situations (6) by generally supporting the key assumptions of TMT; specifically, that mortality salience leads to heightened anxiety levels and compromised well-being, psychological buffers can mitigate this effect, and that the term *terror* (reflecting the impact on anxiety) is justified. Nevertheless, TMT also assumes an impact on other indicators of well-being (physiological and mental health, affect), which we found, but to a much lesser extent, and also assumes that there are protective psychological factors (e.g., self-worth, resilience) that buffer against aversive effects. We found some evidence of habituation to the war situation and some buffering effects, although not for resilience or mental health.

In sum, the present study, in general, supports the assumptions of TMT but additionally ranks the assumptions as follows: (1) impact on anxiety (strongest); (2) mental health and somatic symptom severity; and (3) protective factors (weakest).

## Data availability statement

The datasets presented in this study can be found in online repositories. The names of the repository/repositories and accession number(s) can be found at: https://osf.io/s2ypw/.

## Ethics statement

The studies involving humans were approved by the Research Ethics Committee of the Faculty of Psychology of Taras Shevchenko National University of Kyiv, registration number 11-22/6. The studies were conducted in accordance with the local legislation and institutional requirements. The participants provided their written informed consent to participate in this study.

## Author contributions

SS, DL, and AK contributed to the conception and design of the study. AK and SP recruited participants. DL supervised the technical realisation of the study using ESMira and provided technical support with translations. SS performed the statistical analysis. SS wrote the first draft of the manuscript. AK wrote sections of the manuscript. All authors contributed to the article and approved the submitted version.

## Footnotes

^1^Two participants completed the demographic questionnaire and final questionnaire, but only once the daily questionnaire.

^2^https://esmira.kl.ac.at/

^3^https://air-alarms.in.ua/
