# Supplementary material for: Examining terror management theory in Ukraine: impact of air-raid alarms and explosions on mental health, somatic symptoms, and well-being
Source: Front Psychiatry. 2023 Oct 31;14:1244335. doi: 10.3389/fpsyt.2023.1244335 (PMC10644072; doi:10.3389/fpsyt.2023.1244335)
Supplement: Supplementary file 1 [file Data_Sheet_1.docx]

Supplementary Material

Examining Terror Management Theory in Ukraine: Impact of Air-Raid Alarms and Explosions on Mental Health, Somatic Symptoms, and Well-being

Stefan Stieger^1^, David Lewetz^1^, Svitlana Paschenko^2^, Anton Kurapov^2,3^

^1^ Department of Psychology and Psychodynamics, Karl Landsteiner University of Health Sciences, Krems an der Donau, Austria

^2^ Faculty of Psychology, Taras Shevchenko National University of Kyiv, Kyiv, Ukraine

^3^ Department of Psychology, Faculty of Natural Sciences, University of Salzburg, Salzburg, Austria

*** Correspondence:** Stefan Stieger, [stefan.stieger@kl.ac.at](mailto:stefan.stieger@kl.ac.at)

# Supplementary Figures and Tables

**Figure S1**. Screenshots of graphical feedback.

| Left panel: General feedback over all participants | Right panel: Personalized feedback only for the respective participant |
| --- | --- |
| 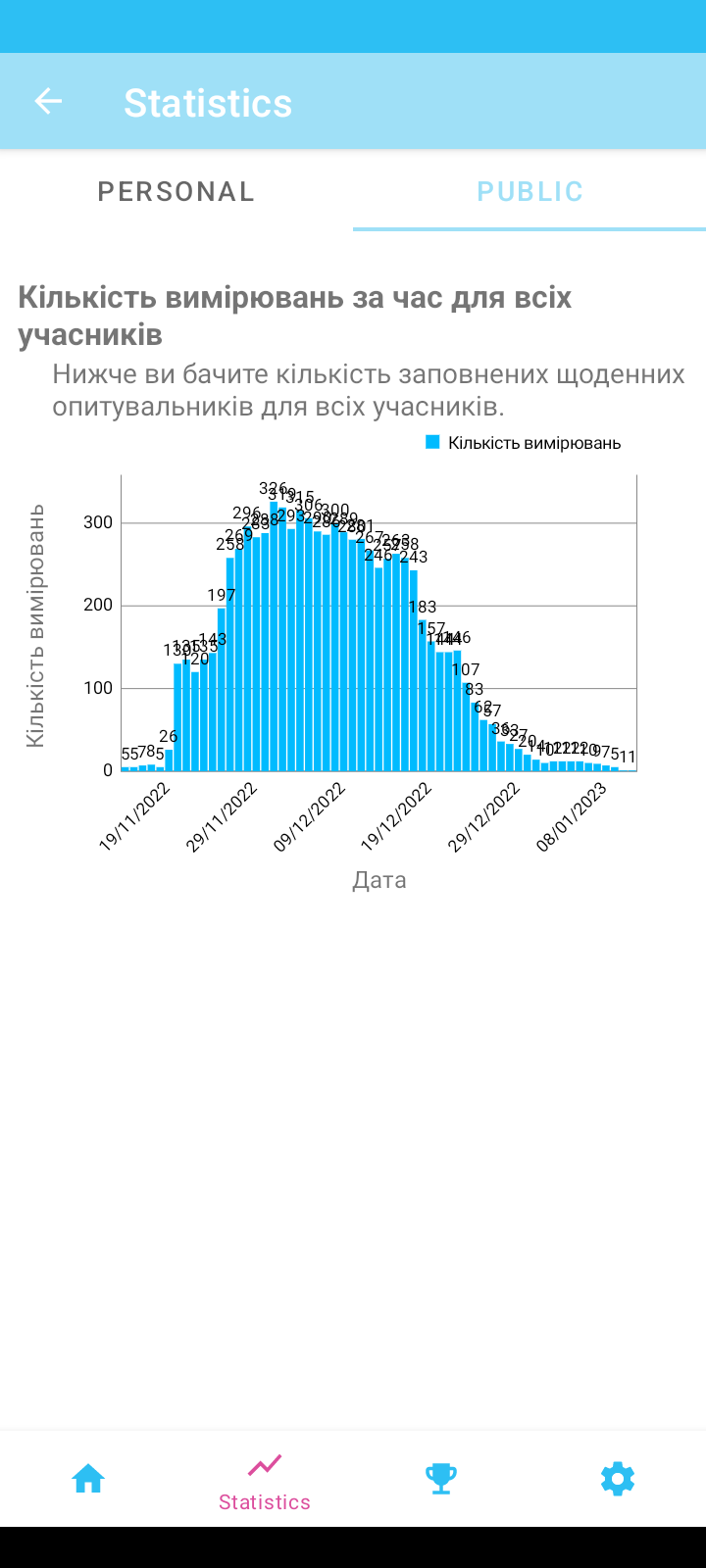 | 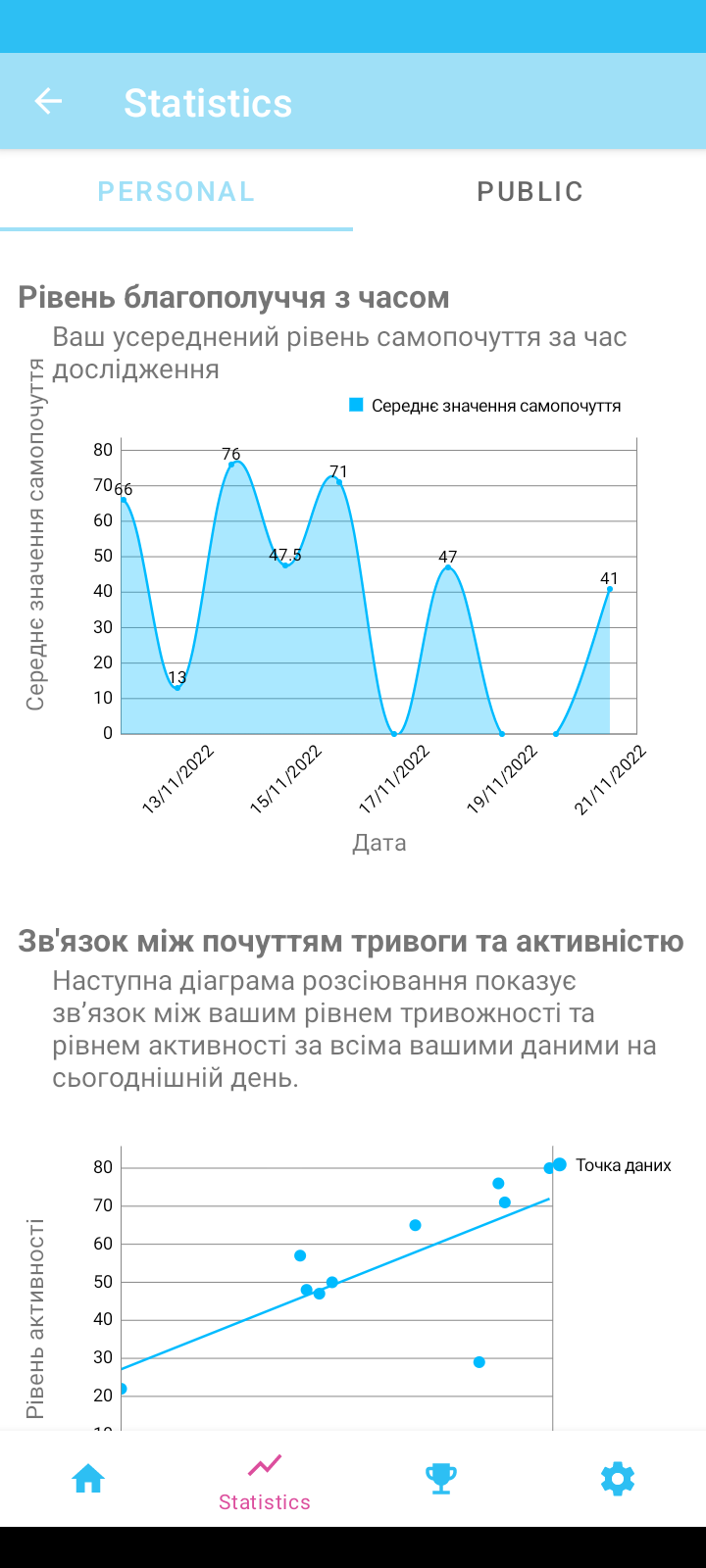 |

**Figure S2**. Interaction between general perceived stress and anxiety based on the number of explosions.


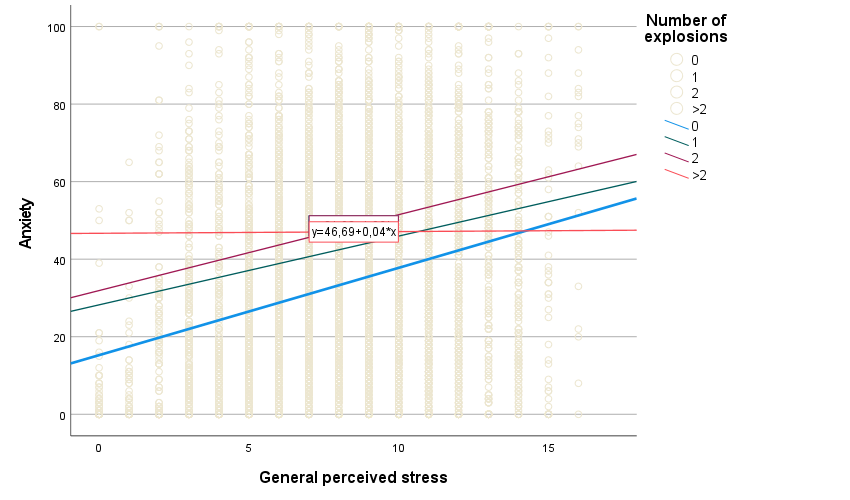


**Figure S3**. Interaction between habituation and anxiety based on the number of explosions (95% loess curves).


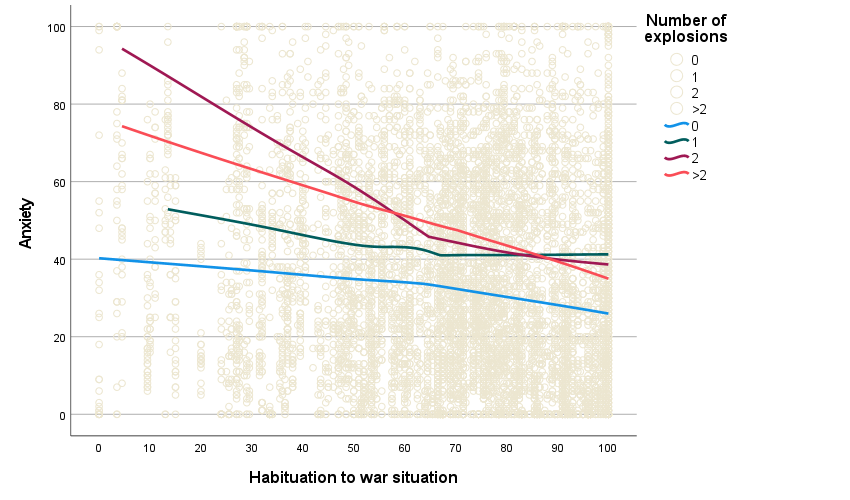


**Table S1**. Reliabilities of Level 2 measures (final questionnaire).

|  | Cronbach α | McDonald ω |
| --- | --- | --- |
| SSS8 | .816 | .818 |
| PSS4 | .778 | .777 |
| BRS | .800 | .806 |
| MHC overall | .951 | .951 |
| Subscale Emotional Well-Being | .888 | .890 |
| Subscale Social Well-Being | .893 | .892 |
| Subscale Psychological Well-Being | .912 | .912 |

*Note*. CI = 95% Confidence Interval, all variables represent the measurement point at the end of the study, PSS = Perceived Stress Scale, SCI = Stress and Coping Inventar, TICS = Trier Inventory for Chronic Stress, SWLS = Satisfaction With Life Scale, HINT = Habit Index of Negative Thinking, SSS–8 = Somatic Symptom Scale-8, RS–11 = Resilience Scale–11

**Table S2**. Reliabilities of Level 1 measures (daily questionnaire).

|  | Within-person *R*_C_ | Between-person *R*_kR_ |
| --- | --- | --- |
| MHC overall | .87  .86 | .98  .98 |
| MHC emotional | .81  .81 | .95  .94 |
| MHC social | NA  .72 | NA  .97 |
| MHC psychological | .76  .74 | .98  .98 |
| SSS8 | .61  .60 | .98  .97 |

*Note*. First line entry = Android (*n* = 3,784 questionnaires); second-line entry = iOS (*n* = 4,040 questionnaires). NA = could not be calculated due to a convergence problem.

**Table S3**. Intercorrelations of study variables (Level 2) for the whole sample as well as sex-specific.

|  | 1 | 2 | 3 | 4 | 5 | 6 | 7 | 8 |
| --- | --- | --- | --- | --- | --- | --- | --- | --- |
| 1 Age |  | .03 | -.03 | .02 | .06 | -.16** | -.11 | -.01 |
| 2 MHC overall | -.13  .08 |  | .89*** | .92*** | .95*** | -.27*** | -.58*** | .26*** |
| 3 MHC Emotional | -.24  .04 | .91***  .89*** |  | .72*** | .84*** | -.26*** | -.60*** | .26*** |
| 4 MHC Social | -.06  .06 | .87***  .93*** | .64***  .74*** |  | .77*** | -.21*** | -.42*** | .18*** |
| 5 MHC Psychological | -.10  .11 | .95***  .95*** | .90***  .82*** | .69***  .79*** |  | -.28*** | -.60*** | .29*** |
| 6 SSS-8 | -.06  -.17* | -.27  -.28*** | -.27  -.26*** | -.16  -.23*** | -.29*  -.28*** |  | .42*** | -.27*** |
| 7 PSS-4 | .16  -.17* | -.67***  -.56*** | -.75***  -.57*** | -.40**  -.43*** | -.72***  -.58*** | .24  .46*** |  | -.45*** |
| 8 BRS | -.17  -.02 | .32*  .26*** | .39**  .24*** | .12  .21** | .38**  .28*** | -.14  -.27*** | -.61***  -.40*** |  |

*Note*. Above diagonal entries = whole dataset (*N* = 284-306). Below diagonal entries separately for male (first line entries; *n* = 45-50) and female (second line entries; *n* = 239-256). *** *p* < .001, ** *p* < .01, * *p* < .05 (two-sided).

**Table S4**. Multilevel models analyzing a possible intervention effect due to the number of graphics viewed during the assessment phase.

|  | Dependent variables (Level 1; evening questionnaire) | | | | |
| --- | --- | --- | --- | --- | --- |
|  | Symptom severity | Mental health | Anxiety | Pleasantness | Arousal |
|  | Standardized effect size β | | | | |
| Predictors |  |  |  |  |  |
| A: Number of graphics viewed | -.05 | -.12** | -.02 | >-.01 | -.07* |
| B: Day of the study | -.11*** | -.02*** | -.04*** | .02 | <.01 |
| Interaction A×B | >-.01 | .02** | -.03** | .02* | <.01 |

*Note*. *** *p* < .001, ** *p* < .01, * *p* < .05 (two-sided).

**Table S5**. Standardized main and cross-level effects (fixed-effects model).

|  |  | MHC | | SSS-8 | | Valence | | Arousal | | Anxiety | |
| --- | --- | --- | --- | --- | --- | --- | --- | --- | --- | --- | --- |
| Predictors |  | β | *CI* | β | *CI* | β | *CI* | β | *CI* | β | *CI* |
| Main effects |  |  |  |  |  |  |  |  |  |  |  |
| (Intercept) |  | -0.18 | -0.41, 0.04 | -0.08 | -0.32, 0.15 | 0.06 | -0.11, 0.24 | -0.11 | -0.29, 0.07 | -0.33 | -0.53, -0.14 |
| Air-raid alarms [1] |  | -0.05 | -0.14, 0.04 | 0.10 | 0.00, 0.20 | -0.03 | -0.17, 0.11 | -0.09 | -0.23, 0.06 | 0.19 | 0.06, 0.32 |
| Air-raid alarms [>1] |  | 0.05 | -0.09, 0.19 | 0.14 | -0.01, 0.29 | -0.02 | -0.22, 0.19 | -0.06 | -0.28, 0.16 | 0.00 | -0.21, 0.20 |
| Explosions [1] |  | 0.14 | -0.05, 0.34 | -0.05 | -0.26, 0.16 | 0.12 | -0.18, 0.42 | 0.07 | -0.23, 0.38 | 0.11 | -0.18, 0.40 |
| Explosions [2] |  | 0.01 | -0.28, 0.30 | 0.15 | -0.16, 0.46 | 0.17 | -0.27, 0.60 | -0.16 | -0.61, 0.30 | 0.28 | -0.14, 0.69 |
| Explosions [>2] |  | -0.14 | -0.32, 0.05 | 0.08 | -0.11, 0.27 | -0.21 | -0.48, 0.07 | -0.14 | -0.42, 0.15 | 0.56 | 0.30, 0.82 |
| Electricity outage [Yes] |  | 0.03 | -0.09, 0.16 | 0.00 | -0.13, 0.13 | -0.11 | -0.28, 0.07 | 0.00 | -0.18, 0.18 | 0.01 | -0.16, 0.19 |
| Heating problem [Yes] |  | 0.05 | -0.08, 0.17 | -0.04 | -0.17, 0.09 | 0.12 | -0.06, 0.30 | -0.04 | -0.23, 0.15 | 0.17 | 0.00, 0.35 |
| Water supply problem [Yes] |  | -0.10 | -0.22, 0.02 | 0.05 | -0.08, 0.18 | -0.12 | -0.30, 0.06 | 0.00 | -0.19, 0.18 | 0.15 | -0.02, 0.33 |
| Day of study |  | -0.01 | -0.02, 0.00 | -0.12 | -0.13, -0.10 | 0.03 | 0.01, 0.05 | 0.01 | -0.01, 0.04 | -0.06 | -0.08, -0.04 |
| Age |  | 0.11 | 0.03, 0.20 | -0.04 | -0.14, 0.05 | 0.07 | 0.00, 0.14 | 0.07 | 0.00, 0.14 | -0.03 | -0.11, 0.04 |
| Sex [Female] |  | 0.28 | 0.03, 0.52 | 0.03 | -0.23, 0.29 | 0.03 | -0.17, 0.23 | 0.17 | -0.02, 0.37 | 0.19 | -0.02, 0.41 |
| PSS-4 |  | -0.41 | -0.51, -0.31 | 0.21 | 0.11, 0.31 | -0.23 | -0.31, -0.15 | -0.14 | -0.22, -0.06 | 0.18 | 0.10, 0.27 |
| BRS |  | 0.00 | -0.10, 0.10 | -0.13 | -0.23, -0.03 | 0.08 | 0.01, 0.16 | 0.07 | 0.00, 0.15 | -0.09 | -0.17, -0.01 |
| Habituation |  | 0.02 | -0.07, 0.11 | 0.00 | -0.10, 0.08 | 0.01 | -0.05, 0.08 | 0.00 | -0.07, 0.07 | -0.10 | -0.17, -0.02 |
| Cross-level interactions | Interaction with |  |  |  |  |  |  |  |  |  |  |
| Explosions [1] | Age | -0.05 | -0.14, 0.04 | -0.02 | -0.11, 0.07 | -0.08 | -0.21, 0.05 | 0.00 | -0.13, 0.14 | 0.00 | -0.13, 0.13 |
| Explosions [1] | Sex [Female] | -0.22 | -0.44, 0.00 | -0.02 | -0.26, 0.22 | -0.31 | -0.65, 0.03 | 0.00 | -0.34, 0.36 | 0.22 | -0.11, 0.56 |
| Explosions [1] | PSS-4 | 0.00 | -0.10, 0.09 | 0.12 | 0.02, 0.23 | -0.02 | -0.17, 0.13 | -0.17 | -0.32, -0.02 | 0.00 | -0.14, 0.14 |
| Explosions [1] | BRS | -0.08 | -0.18, 0.03 | 0.03 | -0.08, 0.14 | -0.11 | -0.27, 0.04 | -0.02 | -0.18, 0.14 | 0.11 | -0.04, 0.26 |
| Explosions [1] | Habituation | 0.09 | 0.00, 0.19 | 0.00 | -0.10, 0.10 | 0.05 | -0.10, 0.20 | 0.03 | -0.13, 0.18 | -0.05 | -0.19, 0.09 |
| Explosions [2] | Age | 0.04 | -0.09, 0.16 | -0.05 | -0.18, 0.08 | -0.04 | -0.23, 0.15 | 0.00 | -0.21, 0.19 | -0.07 | -0.24, 0.11 |
| Explosions [2] | Sex [Female] | -0.09 | -0.41, 0.23 | -0.20 | -0.53, 0.14 | -0.34 | -0.82, 0.14 | 0.00 | -0.50, 0.50 | 0.20 | -0.25, 0.66 |
| Explosions [2] | PSS-4 | -0.02 | -0.16, 0.11 | -0.03 | -0.17, 0.12 | -0.11 | -0.31, 0.10 | -0.15 | -0.36, 0.05 | -0.03 | -0.21, 0.16 |
| Explosions [2] | BRS | -0.08 | -0.22, 0.06 | -0.13 | -0.28, 0.02 | -0.19 | -0.41, 0.02 | -0.10 | -0.32, 0.12 | 0.04 | -0.16, 0.24 |
| Explosions [2] | Habituation | 0.13 | 0.00, 0.25 | -0.12 | -0.25, 0.02 | 0.13 | -0.06, 0.32 | 0.03 | -0.17, 0.23 | **-0.31** | -0.49, -0.13 |
| Explosions [>2] | Age | 0.04 | -0.04, 0.12 | -0.02 | -0.10, 0.07 | 0.07 | -0.05, 0.18 | 0.14 | 0.02, 0.26 | 0.04 | -0.07, 0.15 |
| Explosions [>2] | Sex [Female] | 0.04 | -0.17, 0.24 | 0.01 | -0.21, 0.23 | -0.01 | -0.32, 0.30 | 0.08 | -0.24, 0.40 | -0.05 | -0.34, 0.25 |
| Explosions [>2] | PSS-4 | 0.01 | -0.07, 0.10 | -0.03 | -0.12, 0.06 | 0.11 | -0.01, 0.24 | 0.11 | -0.02, 0.25 | **-0.24** | -0.36, -0.11 |
| Explosions [>2] | BRS | 0.00 | -0.09, 0.09 | -0.04 | -0.13, 0.05 | -0.03 | -0.16, 0.10 | 0.12 | -0.02, 0.25 | -0.03 | -0.16, 0.09 |
| Explosions [>2] | Habituation | 0.03 | -0.06, 0.13 | -0.09 | -0.19, 0.01 | 0.10 | -0.04, 0.25 | 0.09 | -0.06, 0.23 | -0.17 | -0.31, -0.04 |
| Electricity outage [Yes] | Age | 0.03 | -0.02, 0.08 | 0.02 | -0.03, 0.07 | -0.05 | -0.12, 0.02 | -0.01 | -0.08, 0.06 | 0.05 | -0.02, 0.11 |
| Electricity outage [Yes] | Sex [Female] | -0.03 | -0.17, 0.10 | 0.00 | -0.14, 0.15 | 0.14 | -0.05, 0.33 | 0.07 | -0.14, 0.27 | 0.02 | -0.17, 0.21 |
| Electricity outage [Yes] | PSS-4 | 0.00 | -0.06, 0.05 | 0.02 | -0.03, 0.08 | -0.08 | -0.15, 0.00 | -0.04 | -0.12, 0.04 | 0.00 | -0.08, 0.06 |
| Electricity outage [Yes] | BRS | 0.05 | 0.00, 0.10 | 0.00 | -0.06, 0.05 | 0.00 | -0.07, 0.08 | 0.00 | -0.08, 0.07 | -0.02 | -0.09, 0.06 |
| Electricity outage [Yes] | Habituation | 0.00 | -0.06, 0.05 | 0.03 | -0.03, 0.09 | 0.00 | -0.08, 0.08 | -0.03 | -0.11, 0.05 | 0.07 | 0.00, 0.15 |
| Heating problem [Yes] | Age | -0.01 | -0.07, 0.04 | 0.01 | -0.04, 0.07 | -0.02 | -0.10, 0.05 | 0.10 | 0.02, 0.17 | -0.02 | -0.09, 0.06 |
| Heating problem [Yes] | Sex [Female] | -0.15 | -0.29, -0.02 | 0.16 | 0.01, 0.30 | -0.30 | -0.50, -0.10 | -0.06 | -0.26, 0.15 | -0.08 | -0.27, 0.11 |
| Heating problem [Yes] | PSS-4 | 0.00 | -0.04, 0.06 | 0.01 | -0.04, 0.06 | 0.00 | -0.06, 0.08 | 0.01 | -0.06, 0.09 | 0.01 | -0.06, 0.08 |
| Heating problem [Yes] | BRS | -0.01 | -0.07, 0.04 | -0.04 | -0.10, 0.01 | -0.02 | -0.10, 0.06 | 0.00 | -0.09, 0.07 | -0.03 | -0.11, 0.04 |
| Heating problem [Yes] | Habituation | -0.02 | -0.07, 0.04 | 0.04 | -0.02, 0.10 | -0.05 | -0.13, 0.03 | 0.01 | -0.07, 0.10 | -0.05 | -0.12, 0.03 |
| Water supply problem [Yes] | Age | -0.03 | -0.08, 0.02 | 0.02 | -0.03, 0.08 | 0.00 | -0.07, 0.08 | -0.02 | -0.09, 0.06 | 0.02 | -0.05, 0.09 |
| Water supply problem [Yes] | Sex [Female] | 0.11 | -0.03, 0.24 | 0.02 | -0.12, 0.16 | 0.01 | -0.18, 0.21 | -0.12 | -0.32, 0.08 | -0.11 | -0.30, 0.08 |
| Water supply problem [Yes] | PSS-4 | 0.00 | -0.04, 0.06 | 0.05 | 0.00, 0.11 | -0.06 | -0.13, 0.02 | -0.07 | -0.15, 0.01 | 0.02 | -0.05, 0.09 |
| Water supply problem [Yes] | BRS | 0.02 | -0.03, 0.08 | 0.04 | -0.02, 0.10 | -0.05 | -0.13, 0.04 | -0.05 | -0.14, 0.03 | 0.00 | -0.08, 0.07 |
| Water supply problem [Yes] | Habituation | 0.00 | -0.06, 0.05 | -0.02 | -0.08, 0.04 | -0.02 | -0.10, 0.05 | 0.06 | -0.02, 0.14 | 0.03 | -0.05, 0.10 |
| Air-raid alarms [1] | Age | 0.00 | -0.03, 0.04 | -0.02 | -0.05, 0.02 | -0.02 | -0.08, 0.03 | 0.03 | -0.02, 0.09 | **0.09** | 0.04, 0.14 |
| Air-raid alarms [>1] | Age | -0.02 | -0.07, 0.04 | -0.03 | -0.09, 0.03 | 0.00 | -0.08, 0.08 | 0.02 | -0.07, 0.10 | **0.14** | 0.06, 0.21 |
| Air-raid alarms [1] | Sex [Female] | 0.02 | -0.08, 0.12 | -0.05 | -0.16, 0.05 | 0.00 | -0.15, 0.15 | 0.07 | -0.09, 0.22 | -0.01 | -0.15, 0.13 |
| Air-raid alarms [>1] | Sex [Female] | -0.09 | -0.25, 0.06 | 0.00 | -0.17, 0.16 | -0.09 | -0.32, 0.14 | 0.07 | -0.17, 0.31 | 0.25 | 0.03, 0.48 |
| Air-raid alarms [1] | PSS-4 | -0.03 | -0.07, 0.01 | 0.00 | -0.03, 0.05 | 0.00 | -0.06, 0.06 | 0.01 | -0.05, 0.07 | -0.04 | -0.09, 0.02 |
| Air-raid alarms [>1] | PSS-4 | -0.10 | -0.16, -0.03 | 0.01 | -0.05, 0.08 | 0.01 | -0.08, 0.10 | -0.04 | -0.14, 0.06 | 0.03 | -0.06, 0.12 |
| Air-raid alarms [1] | BRS | -0.03 | -0.07, 0.01 | -0.01 | -0.05, 0.03 | 0.00 | -0.05, 0.07 | 0.01 | -0.05, 0.08 | -0.01 | -0.07, 0.05 |
| Air-raid alarms [>1] | BRS | -0.09 | -0.15, -0.03 | 0.03 | -0.04, 0.09 | -0.01 | -0.11, 0.08 | 0.08 | -0.02, 0.18 | 0.12 | 0.04, 0.21 |
| Air-raid alarms [1] | Habituation | 0.00 | -0.05, 0.03 | -0.01 | -0.06, 0.03 | 0.01 | -0.05, 0.07 | 0.00 | -0.07, 0.06 | -0.04 | -0.10, 0.02 |
| Air-raid alarms [>1] | Habituation | -0.02 | -0.08, 0.05 | -0.09 | -0.15, -0.02 | 0.04 | -0.05, 0.13 | -0.10 | -0.20, 0.00 | -0.04 | -0.13, 0.05 |

Note. MHC = Mental Health Continuum (i.e., psychological health), SSS-8 = Somatic Symptom Scale – 8 (i.e., physiological health), CI = 95% Confidence Interval, β = standardized *B*, PSS-4 = general Perceived Stress Scale – 4, BRS = Brief Resilience Scale. Bold values are significant at Bonferroni corrected *p*-value of *p* < .00125.

**Table S6**. Results of the multilevel analyses to test if mental health acts as a buffer between war specific situations and anxiety.

| Main effects: Standardized β-values |  | Model 1: Without mental health measure | Model 2: With mental health measure |
| --- | --- | --- | --- |
| (Intercept) |  | **-.17*** | **-.15*** |
| Air-raid alarms [1] |  | **.18*** | **.17*** |
| Air-raid alarms [>1] |  | **.22*** | **.21*** |
| Explosions [1] |  | **.29*** | **.28*** |
| Explosions [2] |  | **.43*** | **.43*** |
| Explosions [>2] |  | **.51*** | **.48*** |
| Electricity outage [Yes] |  | .03 | .03 |
| Heating problem [Yes] |  | **.11*** | .08* |
| Water supply problem [Yes] |  | .06 | .06 |
| Day of study |  | **-.07*** | **-.07*** |
| Resilience |  | **-.18*** | **-.18*** |
| Habituation |  | -.10* | -.10* |
| Mental health |  |  | **-.21*** |
| Interactions | Interaction with |  |  |
| Explosions [1] | Resilience | .07 | .05 |
| Explosions [1] | Habituation | -.04 | .01 |
| Explosions [1] | Mental health |  | -.12* |
| Explosions [2] | Resilience | .04 | .03 |
| Explosions [2] | Habituation | **-.30*** | **-.28*** |
| Explosions [2] | Mental health |  | .06 |
| Explosions [>2] | Resilience | .08 | .07 |
| Explosions [>2] | Habituation | -.17* | -.16* |
| Explosions [>2] | Mental health |  | >-.01 |
| Electricity outage [Yes] | Resilience | >-.01 | >-.01 |
| Electricity outage [Yes] | Habituation | .06 | .06 |
| Electricity outage [Yes] | Mental health |  | .02 |
| Heating problem [Yes] | Resilience | -.04 | -.04 |
| Heating problem [Yes] | Habituation | -.03 | -.03 |
| Heating problem [Yes] | Mental health |  | .05* |
| Water supply problem [Yes] | Resilience | >-.01 | >-.01 |
| Water supply problem [Yes] | Habituation | .02 | .01 |
| Water supply problem [Yes] | Mental health |  | .04 |
| Air-raid alarms [1] | Resilience | >-.01 | >-.01 |
| Air-raid alarms [1] | Habituation | -.06* | -.06* |
| Air-raid alarms [1] | Mental health |  | .03 |
| Air-raid alarms [>1] | Resilience | .08* | .07 |
| Air-raid alarms [>1] | Habituation | -.10* | -.10* |
| Air-raid alarms [>1] | Mental health |  | .02 |

*Note.* * *p* < .05; Bold values represent still significant values when applying a Bonferroni corrected *p*-value of *p* < .002 (25 tests).
